# Digitally Delivered Cognitive Behavioral Interventions for Alcohol and Other Drug Use: Meta-Analysis Across Consumption and Psychosocial Outcomes

**DOI:** 10.2196/82370

**Published:** 2026-05-19

**Authors:** Brian D Kiluk, Lara A Ray, Omeed Tartak, Lovisa Werner, Thomas A Trikalinos, Molly Magill

**Affiliations:** 1 Department of Psychiatry Yale School of Medicine New Haven, CT United States; 2 Department of Psychology University of California at Los Angeles Los Angeles, CA United States; 3 Center for Alcohol and Addiction Studies Brown University Providence, RI United States

**Keywords:** alcohol treatment, substance use disorders, cognitive behavioral therapy, digital therapeutics, technology-based treatment, meta-analysis

## Abstract

**Background:**

Cognitive behaviorally based interventions have broad appeal and potential for impact when treating adult alcohol and other drug use. Digitally delivered cognitive behaviorally based interventions (dCBIs) may offer this impact with the benefit of increased accessibility. Although prior reviews have indicated the benefits of dCBIs on substance use outcomes, the extension to psychosocial functioning outcomes is unknown.

**Objective:**

This meta-analysis provides an overview of dCBI effects across a range of functional end points.

**Methods:**

A literature search was conducted through October 2024. All primary and secondary reports of clinical trials of dCBI were obtained, and all available study end points were eligible for meta-analysis. Descriptive data were extracted and categorized into 1 of 13 different outcome types (eg, abstinence, quantity, cognitive, and quality of life) and into 2 broader outcome classes (ie, consumption and psychosocial). Robust variance estimation was used to conduct hypothesis tests on random effects pooled estimates with outcome class and comparison type as the primary subgroup variables of interest.

**Results:**

The study sample included 65 randomized trials (*K*=110 publications; 753 effect sizes) of dCBI for adult alcohol and other drug use. With respect to efficacy, dCBI as a stand-alone treatment in contrast to a minimal treatment control showed positive and statistically significant effects for consumption (*g*=0.27; *P<*.001; *I*^2^=85.1%; *k*=31; *k_es_*=134) and psychosocial (*g*=0.16; *P*=.008; *I*^2^=75.2%; *k*=16; *k_es_*=60) outcomes. As an addition to usual care, efficacy was demonstrated for consumption (*g*=0.23; *P<*.001; *I*^2^=9.8%; *k*=20; *k_es_*=65), but not psychosocial functioning. Efficacy compared to another digital or in-person intervention or cognitive behaviorally based intervention delivered by a therapist was not observed. Within the dCBI condition, large effect sizes were observed for both outcome classes (ie, 60%-80% of participants showed improvement relative to baseline), and effect size magnitude and statistical heterogeneity varied by the type of outcome examined.

**Conclusions:**

These results show a benefit for dCBI as a stand-alone therapy and an addition to usual care. Importantly, stand-alone effects were observed for both consumption and some psychosocial outcomes. This study is the first to offer a comprehensive look at dCBI intervention effects across a range of functional end points.

## Introduction

### Background

Alcohol and other drug use (AOD) disorders are associated with a significant public health burden, including mortality, economic burden, and adverse social- and individual-level consequences [1,2]. They are highly comorbid with other mental health disorders [3] and can have profound negative effects on psychosocial functioning, including overall mental health and quality of life [4]. Although vastly underused, there are a number of effective treatments available for AOD. Among them, cognitive behaviorally based interventions (CBIs) are the most widely researched and clinically practiced interventions for AOD. CBIs (ie, cognitive behavioral therapies or cognitive behavioral therapies with elements of other evidence-based modalities such as motivational interviewing) consist of a broad range of behavioral interventions, including those targeting operant learning processes, cognitive and motivational strategies, and skills training targeting interpersonal, emotion regulation, and organizational or problem-solving deficits [5]. Multiple meta-analyses have provided evidence supporting CBI efficacy in treating AOD [6], including when combined with pharmacotherapy [7], and for co-occurring disorders [8].

Despite its demonstrated efficacy, barriers such as limited access to trained providers, costs of training, geographic limitations, and stigma impede the widespread delivery and uptake of CBIs in traditional settings [9,10]. To overcome these challenges, digital platforms (ie, computer-delivered, web-based, or mobile platforms) have emerged as a promising modality for expanding access to evidence-based care. Given its structured, problem-focused, and time-limited format, CBIs are among the most common therapeutic approaches in digital mental health interventions [11]. These digitally delivered cognitive behaviorally based interventions (dCBIs) aim to replicate the core components of CBIs in a highly scalable and often self-guided format. A body of research has emerged over the past 2 decades indicating support for dCBIs (also referred to as internet-based cognitive behavioral therapy [CBT] and technology-delivered CBT) in treating AOD. Systematic reviews and meta-analyses indicate a small, significant effect of dCBIs on reducing substance use compared to control conditions [12-14]. However, the impact of these interventions on outcomes beyond substance use is relatively unknown. Of particular interest are indicators of cognitive, behavioral, psychological, and lifestyle changes that are targeted by dCBIs that may promote recovery from AOD [15]. Evidence of a positive effect on psychosocial outcomes (eg, mental health, emotional well-being, coping strategies, daily functioning, and quality of life) would enhance the clinical significance of changes in substance use.

### Meta-Analysis Purpose and Aims

The aim of this meta-analysis is to evaluate dCBI effects across 2 broad classes of outcomes for AOD: substance use consumption and psychosocial functioning. These outcome classes were the primary subgroup variable in this study. Because the experimental comparator is a common predictor of effect size variability in meta-analyses [16], the comparator type was the second primary subgroup variable of interest. As a follow-up and in cases of significant statistical heterogeneity within subgroups, regression-based moderator analyses were undertaken. Additional sensitivity analyses were conducted (eg, risk of bias assessment and publication bias assessment) and are reported following the PRISMA (Preferred Reporting Items for Systematic Reviews and Meta-Analyses) guidelines.

## Methods

### Primary Study Literature Search

A librarian-supported literature search was conducted through October 2022 (with an updated search to October 2024) to identify studies for a meta-analysis project on CBIs for AOD. Specifically, a title, abstract, and keyword search by treatment (eg, “cognitive behavioral therapy”), outcome (eg, “alcohol”), and methodological (eg, “randomized clinical trial”) terms was conducted in the PubMed database (sample search strategy can be found in Multimedia Appendix 1). Other databases that were searched included Cochrane CENTRAL and EBSCO (ie, CINAHL, APA PsycINFO, and SocINDEX). Abstract screening occurred in Covidence and was conducted by 4 raters, with 2 raters (OT and LW) for each study, and consensus decisions were made by the lead study investigator (MM). Because varying modes of CBI delivery (eg, delivered via technology, delivered in-person, and combined with other behavioral modalities) were of interest to the larger project, the literature search yielded 1387 primary and secondary clinical trial reports that were downloaded and reviewed for eligibility in 1 or more meta-analyses. Additional steps included a bibliographic review of relevant review papers [17-30] and a clinical trial number search to derive all available publications on a given primary study sample.

### Primary Study Inclusion

Study inclusion criteria were as follows: (1) English language publication, (2) peer-reviewed publication, (3) publication date between 1970 and 2024, (4) randomized controlled trial, (5) adult patient population (median age ≥18 years), (6) patient population meeting criteria for an alcohol or other substance use disorder (ie, Diagnostic and Statistical Manual [DSM] III-R through 5 [31-34]) or at-risk use based on an established criterion (eg, Alcohol Use Disorder Identification Test score >8 [35]), and (7) a cognitive behavioral or combined cognitive behavioral (ie, combined with elements of other evidence-based AOD treatments) intervention delivered in a digital and/or mobile health format (ie, via a computer software program, an internet site, or a mobile phone–based app; see Multimedia Appendix 2 [36-100] for details on dCBI interventions).

### Primary Study Characteristic Variables

There were several study descriptors or moderator variables of interest to this meta-analysis. Because the type of comparator is often a primary driver of effect size magnitude [6,16], effect sizes were pooled overall and by the following 4 between- and 1 within-condition comparator type, respectively: (1) dCBI at follow-up compared to assessment only or minimal treatment (eg, a pamphlet or a noninteractive website with AOD information) at follow-up, (2) dCBI at follow-up compared to another treatment modality (eg, other online intervention and a control treatment) or treatment as usual at follow-up, (3) dCBI added to treatment as usual at follow-up compared to treatment as usual at follow-up, (4) dCBI at follow-up compared to CBI delivered by a counselor at follow-up, and (5) dCBI at follow-up compared to dCBI at baseline. Study characteristic variables were organized by demographic, clinical, treatment, and study method factors. Demographic variables included mean age of participants, percentage of female participants, percentage of participants belonging to 1 or more racial groups (eg, Asian, Black, and White), percentage of participants belonging to the Latine ethnic group, percentage of participants identifying a sexual orientation other than heterosexual, percentage of participants identifying as transgender or other non-cisgender identities, percentage of participants with some college education, and percentage of participants who are full- or part-time employed. Clinical variables included the primary drug targeted (ie, alcohol, cannabis, opioid, polydrug, stimulant), substance use severity (ie, inclusion of individuals with use disorder vs at-risk consumption), and 1 or more co-occurring conditions (eg, depression and pain) as a sample inclusion criterion (ie, yes vs no). Treatment variables were dCBI format (ie, CBI only vs CBI combined with other evidence-based treatment elements), dCBI combined with pharmacotherapy (ie, yes vs no), clinically supported dCBI (ie, yes vs no), and treatment length (ie, number of content modules). Study methodological factors included study sample size, publication year, context (ie, community sample, specialty substance use or mental health clinic, other setting; eg, primary care clinic and college campus), publication country (ie, United States and other countries), and study-level risk-of-bias score [101]. Data extraction guidelines were detailed in a study codebook available, upon request, from the last author (MM). Data were extracted using consensus methods between the third and fourth authors (OT and LW; agreement rate 79.9%). The last author resolved disagreements, with input from members of the full study team as needed.

### Effect Size Calculation and Outcomes of Interest

Hedges *g* is a standardized mean difference with a correction for a slight upward bias in the estimated effect size when samples are small [102].

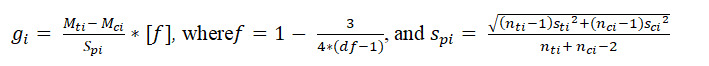



where *i* indexes a trial, *t* and *c* are experimental and control arms, *s* is SDs, *M* is outcome means, *df* is degrees of freedom, and *n* is sample sizes.

The project literature search sought all publications related to a given primary study sample. The purpose of this was to extract all available outcome data points and thus inform a comprehensive picture of dCBI efficacy, effects, and recovery. During the data extraction phase, all available outcomes and their exact measures were identified and grouped into 1 of 13 outcome classes (ie, abstinence, use frequency, heavy use frequency, peak consumption, quantity, dependence severity, other drug use, consequences, cognitive outcomes, coping outcomes, mental health symptoms, general health, and quality of life; see Multimedia Appendix 3 for the full list of study measures and harmonization procedures by outcome class). As a result of this procedure, each primary study could contribute multiple effect sizes, including by comparison type, outcome class, and follow-up time point (ie, the latest data point within 3 ranges: 0-3 months, 4-6 months, and 7 months or later). Effect sizes were reverse-scored as needed (eg, number of days drank) such that a positive effect size indicated a positive treatment outcome for dCBI. Finally, when data from publications were insufficient for effect size calculation (ie, missing variance estimates and statement of “no significant difference between conditions”), raw data were sought from authors (response rate 60%, with 1 study removed due to nonresponse to data request [103]).

### Effect Size Pooling and Sensitivity Analysis

Effect sizes were pooled using inverse variance weighting and a random effects model. Inverse variance weighting allows larger sample studies more weight in the overall pooled effect estimate [104]. A random effects model assumes a distribution, rather than a single population effect size [105]. For hypothesis testing, sources of dependency from multiple study-level effect sizes (ie, multiple study comparators, outcomes, and/or time points) were handled via robust SEs. Specifically, an approximated covariance matrix is derived from the product of within-study residuals and used to calculate cluster-robust SEs and CIs under a *t*-distribution [106]. The within-study correlation between dependent effects was assumed to be constant (ie, default ρ of 0.8), and sensitivity analyses were conducted that varied the correlation value. Cluster-robust SEs also gain accuracy as the sample of primary studies increases, but a small-sample correction allows for stable estimates in samples as small as 10 primary studies [107]. Meta-analysis was performed in the *robumeta* package for R (R Foundation for Statistical Computing) [106].

The random effects and pooled effect sizes in this study were calculated for 2 outcome classes: consumption (ie, abstinence, use frequency, heavy use frequency, peak consumption, quantity, dependence severity, and other drug use) and psychosocial functioning (ie, consequences, cognitive outcomes, coping outcomes, mental health symptoms, general health, and quality of life). These outcome classes as well as comparator type were the primary subgroup moderators of interest. However, in cases of significant residual study heterogeneity (ie, ≥50% [105] of between-study variance relative to sampling variance measured via the *I*^2^ estimate and *k*≥10 [108]), regression-based moderator analyses by time point (ie, posttreatment to 3 months vs 4 months or later) and primary drug targeted (ie, alcohol, polydrug, and other) were undertaken in attempt to explain additional systematic variation between studies. Prediction intervals were additionally calculated in the case of statistical heterogeneity to obtain a range of potential future effect sizes that incorporate the systematic variability in the data [109]. We considered these sensitivity analyses as a method for examining the validity of our a priori subgroups. Additional sensitivity analysis included effect size pooling for each of the 13 outcome types, analysis of influential studies (ie, studies that, if removed, would change the substantive interpretation of the effect size), and analyses using traditional meta-analytic methods (ie, average dependent effect sizes to the study level and then calculate random effects pooled effect sizes and corresponding hypothesis test statistics). To test for potential publication bias, the relationship between the inverse of the SE and effect size was examined with graphical plots and a rank-order correlation [110]. In this analysis, asymmetry within the plot (ie, the majority of studies located at the bottom right quadrant) and a negative correlation would suggest publication bias. Finally, the risk of bias results is summarized for the sample of primary studies.

## Results

**Primary Study Inclusion Results**

**Figure 1:**
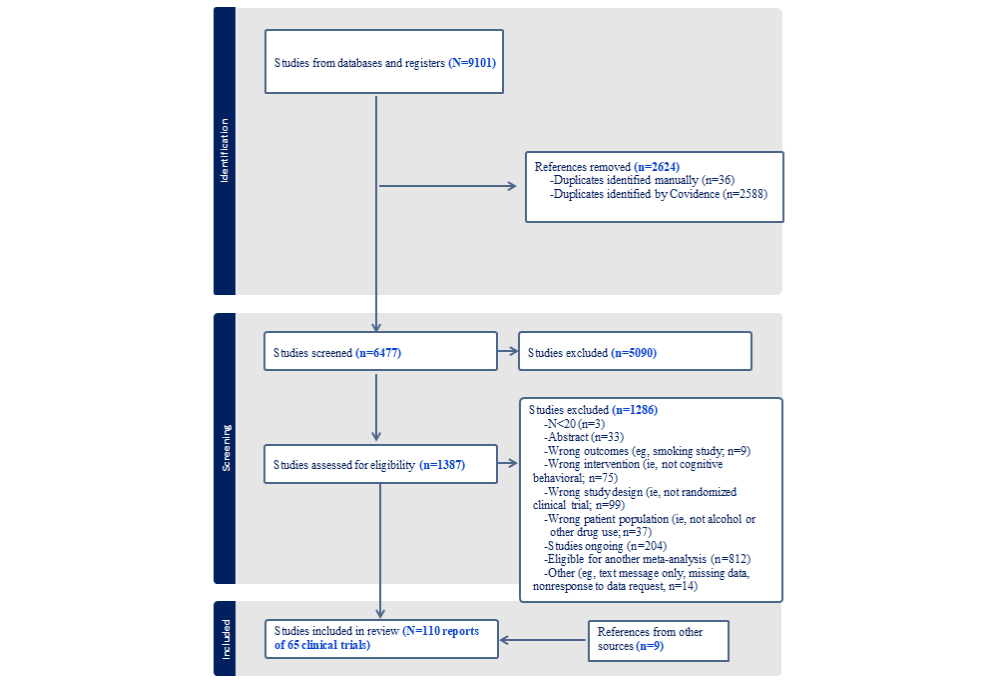
PRISMA (Preferred Reporting Items for Systematic Reviews and Meta-Analyses) flowchart.

PRISMA guidelines were followed (Multimedia Appendix 4), and the study inclusion flow for this study is summarized in Figure 1. The final meta-analytic sample included *K*=65 studies (*k*=45 related reports; N=24,145 individuals) of dCBIs for AOD.

### Primary Study Descriptive Characteristics

All sample-level study characteristics are summarized in Table 1, while characteristics at the individual study level are in Table 2. The sample included 65 randomized trials (*K*=110 publications; 753 effect sizes [consumption: 475; psychosocial: 278]) of dCBI for adult AOD published between 1997 and 2024 [36-44,46-100,111-150]. The mean sample size was 371.46 (SD 997.65) participants with a minimum of 20 [36] and a maximum of 7935 [37]. For participant characteristics, the samples’ mean age was 37.69 (SD 8.88) years, and samples were 45.63% female (SD 19.89%; 100% for 2 studies [38,39]) on average. Among studies that reported racial and ethnic data, the mean samples were 21.77% Asian or Pacific Islander (SD 38.92%; 100% Asian in Liang et al [111]), 34.24% (SD 24.11%) Black, 15.49% (SD 28.67%) Native American or Indigenous person, 59.62% (SD 24.43%) White, and 6.99% (SD 5.84%) multiracial. The mean samples were 20.25% (SD 22.79%; 100% in Paris et al [40]) of Latine ethnicity. Additional sample characteristics included sexual orientation, sex identity, education, and employment status. While reporting on these factors was relatively infrequent across studies, these descriptive data are summarized in Table 1.

**Table 1:** Meta-analysis sample study characteristics.

Variable		*K*^a^ (%)	Mean (SD)	Studies (n=65), n (%)	
Study		65 (100)	371.46 (997.65)	—^b^	
Study date		65 (100)	2016.58 (4.86)	—	
Age (years)		63 (97)	37.69 (8.88)	—	
**Sex**					—
	Female	65 (100)	45.63 (19.89)		
	Transgender	4 (6)	3.93 (3.35)		
	Not heterosexual	3 (5)	26.93 (11.90)		
**Race and ethnicity**					—
	Asian or Pacific Islander	6 (9)	21.77 (38.92)		
	Black	20 (31)	34.24 (24.11)		
	Hispanic or Latine	22 (34)	20.25 (22.79)		
	Indigenous	7 (11)	15.49 (28.67)		
	White	25 (38)	59.62 (24.43)		
	Multiracial	8 (12)	6.99 (5.84)		
**Education**					
	Some college	23 (35)	45.81 (23.69)	—	
**Employment status**					
	Employed	38 (58)	50.81 (22.17)	—	
**Drug targeted**		—	—		
	Alcohol			35 (53.85)	
	Cannabis			7 (10.77)	
	Opioid			5 (7.69)	
	Polydrug			13 (20)	
	Stimulant			5 (7.69)	
**Use disorder**		—	—		
	No			25 (38.46)	
	Yes			40 (61.54)	
**Co-occurring condition**		—	—		
	No			55 (84.62)	
	Yes			10 (15.38)	
**Combined therapy**		—	—		
	MI^c^ alone			27 (41.54)	
	Other			12 (18.46)	
	Not applicable			26 (40)	
**Plus pharmacotherapy**		—	—		
	No			52 (80)	
	Yes			13 (20)	
**Clinician supported**		—	—		
	No			41 (63.08)	
	Yes			24 (36.92)	
Number of modules		54	12.20 (15.17)	—	
**Context**		—	—		
	College setting			4 (6.15)	
	Community sample			30 (46.15)	
	Medical setting			2 (3.08)	
	Other or multiple settings			12 (18.46)	
	Specialty setting			17 (26.15)	
**Publication country**		—	—		
	United States			26 (40)	
	Non–United States			39 (60)	
**Overall risk of bias**		—	—		
	High risk			53 (81.54)	
	Low risk			1 (1.54)	
	Unclear			11 (16.94)	

^a^K=number of studies.

^b^Not applicable.

^c^MI: motivational interviewing.

**Table 2:** Digitally delivered cognitive behaviorally based intervention (dCBI) primary study characteristics at the study level^a^.

Author (year)			Values, n		Treatment and comparison conditions		Drug targeted		Time point		Outcomes
**dCBI compared to assessment only or minimal treatment**											
	Andersson (2015) [47]	1678		Internet intervention versus no treatment		Alcohol		6 weeks		Use frequency, peak consumption, quantity, dependence severity	
	Baumgartner et al (2021) [49]	689		Adherence-focused guidance enhancement for alcohol and depression versus internet as usual		Alcohol		3, 6 months		Abstinence, use frequency, quantity, dependence severity, mental health symptoms	
	Baumgartner et al (2021) [50]	575		CANreduce 2 versus internet as usual		Cannabis		3 months		Use frequency, dependence severity, mental health symptoms	
	Blankers et al (2011) [52], arm 1	205		Self-help alcohol online versus waitlist		Alcohol		3, 6 months		Quantity, dependence severity, quality of life	
	Bonar et al (2022) [53]	149		Social media intervention versus attention-matched Facebook group		Cannabis		3, 6 months		Use frequency, quantity	
	Brendryen et al (2014) [54]	244		Balance versus screening with feedback plus online booklet		Alcohol		2, 6 months		Quantity	
	Brief et al (2013) [41]	600		VetChange versus delayed intervention group		Alcohol		0 weeks		Heavy use frequency, quantity, mental health symptoms	
	Chander et al (2021) [38]	439		Computer-delivered brief alcohol intervention versus oral health intervention		Alcohol		3, 6, 12 months		Use frequency, heavy use frequency, quantity	
	Cunningham (2012) [43]	170		The alcohol help center versus Check Your Drinking screener		Alcohol		Posttreatment, 6 months		Peak, quantity, dependence severity	
	Daros et al (2024) [62]	72		Pocket Skills 2.0 versus delayed treatment		Polydrug		4, 12 weeks		Severity, coping outcomes, mental health symptoms, quality of life	
	Deady et al (2016) [42]	104		DEAL Project versus HealthWatch attention-matched control		Alcohol		3, 6 months		Use frequency, quantity, mental health symptoms	
	Gajecki et al (2017) [64]	186		TeleCoach versus waitlist		Alcohol		12 weeks		Quantity	
	Guillemont et al (2017) [68]	1147		Alcoometre versus brief feedback with diary		Alcohol		0 weeks		Quantity, dependence severity	
	Hester and Delaney (1997) [36]	40		Behavioral Self-Control Program for Windows versus waitlist		Alcohol		0 weeks		Use frequency, peak consumption, quantity	
	Johansson et al (2021) [72]	1169		Self-help internet-based CBT^b^ versus information on changing alcohol habits		Alcohol		3, 6 months		Abstinence, peak consumption, quantity, dependence severity, mental health symptoms, quality of life	
	Kramer et al (2009) [78]	181		Drinking Less TV self-help course versus waitlist		Alcohol		0 weeks		Quantity, dependence severity, consequences	
	Leeman et al (2016) [79]	208		Tertiary Health Research Intervention via Email versus Electronic brochure		Alcohol		1, 6 months		Use frequency, heavy use frequency, peak consumption, quantity, consequences, coping outcomes	
	Liang et al (2018) [111]	75		S-Health versus psychoeducational text messages alone		Opioids		4 weeks		Abstinence, use frequency	
	Mujcic et al (2022) [81]	103		MyCourse versus digital brochure		Alcohol		2, 5, 11 months		Abstinence, quantity, dependence severity	
	Riper et al (2008) [84]	261		Drinking Less versus psychoeducational brochure		Alcohol		6 months		Quantity, dependence severity	
	Schaub et al (2015) [87]	308		CANreduce versus waitlist		Cannabis		3 months		Use frequency, quantity, dependence severity, other drug use, mental health symptoms	
	Schaub et al (2019) [86]	311		Snow control versus waitlist		Stimulant		Posttreatment, 6 months		Use frequency, quantity, dependence severity, other drug use, mental health symptoms	
	Sinadinovic et al (2014) [91], arm 1	633		Alkoholhjalpen versus waitlist		Alcohol		3, 6, 12 months		Quantity	
	Sinadinovic et al (2020) [90]	303		A way out of fog versus waitlist		Cannabis		6 weeks		Abstinence, quantity, dependence severity, other drug use, mental health symptoms, quality of life	
	Stapinski et al (2021) [92]	123		Inroads anxiety and alcohol use intervention versus assessment plus alcohol information		Alcohol		3 weeks, 5 months		Peak, quantity, dependence severity, consequences, mental health symptoms, quality of life	
	Sunami et al (2021) [93]	100		Sensible and Natural Alcoholism Prevention Program for You: Diary On Computer versus assessment only		Alcohol		8 weeks		Abstinence, quantity, dependence severity	
	Sundström et al (2020) [94]	166		Low-intensity internet intervention versus waitlist		Alcohol		0 weeks		Heavy use frequency, quantity, dependence severity, cognitive outcomes, mental health symptoms, quality of life	
	Tait et al (2014) [95]	160		breakingtheice versus waitlist		Stimulant		6 months		Abstinence, consequences, other drug use, cognitive outcomes, mental health symptoms, quality of life	
	Wallace et al (2011) [37]	7935		Down Your Drink versus text-based information about alcohol harms		Alcohol		3, 12 months		Use frequency, peak consumption	
	Wilks et al (2018) [99]	59		Internet-delivered DBT^c^ skills training intervention versus Waitlist		Alcohol		2 months		Quantity, dependence severity, mental health symptoms	
	Zill et al (2019) [100]	608		Vorvida versus waitlist		Alcohol		0 weeks		Peak, consequences, quantity	
**dCBI compared to another treatment or TAU^d^**											
	Augsburger et al (2022) [48]	589		Online self-help intervention versus self-test including personalized feedback		Alcohol		6 months		Abstinence, quantity, dependence severity, cognitive outcomes, mental health symptoms	
	Berman et al (2020) [51]	89		TeleCoach versus control app		Alcohol		6 weeks		Use frequency, peak consumption, quantity, cognitive outcomes	
	Blankers et al (2011) [52], arm 2	205		Self-help alcohol online versus therapy alcohol online		Alcohol		3, 6 months		Quantity, dependence severity, quality of life	
	Budney et al (2015) [55], arm 1	75		Computer-delivered MET^e^/CBT/CM^f^ (COMPUTER) versus brief MET intervention+incentive program		Cannabis		Posttreatment, 9 months		Abstinence	
	Gonzalez and Dulin (2015) [66]	60		Location-based monitoring and intervention for alcohol use disorders versus Drinker’s Check-up plus bibliotherapy		Alcohol		0 weeks		Abstinence, heavy use frequency	
	Kay-Lambkin et al (2009) [75], arm 1	97		Computer-delivered Self-Help for Alcohol and other drug use and Depression versus brief intervention alone		Polydrug		3, 9 months		Heavy use frequency, mental health symptoms	
	Kay-Lambkin et al (2011) [74], arm 1	274		Clinician-assisted computerized treatment versus person-centered therapy		Polydrug		Posttreatment, 1, 4, 10 months		Severity, mental health symptoms, other	
	Kiluk et al (2018) [77], arm 1	137		CBT4CBT^g^ versus Outpatient TAU		Polydrug		Posttreatment, 3, 6 months		Abstinence, dependence severity, frequency, consequences	
	O’Donnell et al (2019) [82]	45		Minimize versus control app with self-monitoring only		Alcohol		0 weeks		Peak, coping outcomes, general health, quality of life	
	Olthof et al (2023) [83]	378		ICan versus online educational modules		Cannabis		3, 6 months		Use frequency, quantity, dependence severity, cognitive outcomes, coping outcomes	
	Rooke et al (2013) [85]	225		Reduce Your Use versus control information		Cannabis		3 months		Abstinence, use frequency, quantity, dependence severity, consequences	
	Schaub et al (2012) [88]	196		Snow control versus psychoeducational modules		Stimulant		Posttreatment, 20 weeks		Abstinence, quantity, dependence severity, other drug use, cognitive outcomes, mental health symptoms	
	Sinadinovic et al (2014) [91], arm 2	633		Alkoholhjalpen versus eScreen.se		Alcohol		3, 6, 12 months		Quantity	
**dCBI plus TAU compared to TAU only**											
	Acosta et al (2017) [46]	162		Thinking Forward+TAU versus primary care TAU		Polydrug		3 months		Use frequency, heavy use frequency, other drug use, mental health symptoms, quality of life	
	Campbell et al (2014) [56]	507		TES^h^+TAU versus TAU		Polydrug		0 weeks		Abstinence	
	Campbell et al (2023) [57]	53		TES-Native version+TAU versus TAU		Polydrug		0, 12 weeks		Abstinence, consequences, coping outcomes, quality of life	
	Carroll et al (2008) [58]	73		CBT4CBT+TAU versus TAU		Polydrug		Posttreatment, 3, 6 months		Abstinence, coping outcomes	
	Carroll et al (2014) [59]	101		CBT4CBT+TAU versus TAU (MMT^i^)		Stimulant		Posttreatment, 6 months		Abstinence, consequences, coping outcomes	
	Carroll et al (2018) [60]	120		CBT4CBT+TAU versus TAU (MMT)		Stimulant		Posttreatment, 3 months		Abstinence, quantity	
	Christensen et al (2014) [61]	170		CRA^j^+CM online versus buprenorphine+CM		Opioids		0 weeks		Abstinence	
	Farren et al (2015) [63]	55		Cognitive relapse prevention-based computerized therapy+TAU versus attention-matched arithmetic exercises+TAU		Alcohol		3 months		Abstinence, use frequency, quantity, cognitive outcomes, mental health symptoms	
	Glasner et al (2020) [65]	35		CBT intervention (ALC^k^-TXT^l^-CBT)+TAU versus informational pamphlet and HIV TAU		Alcohol		0 weeks		Use frequency, heavy use frequency, general health	
	Guarino et al (2018) [67]	110		Take Charge of Pain+TAU versus pain practice care TAU		Opioids		3 months		Consequences, general health, coping outcomes, mental health symptoms, quality of life	
	Hester et al (2011) [69]	84		ModerateDrinking+Moderation Management versus Moderation Management alone		Alcohol		3, 6, 12 months		Consequences, quantity	
	Hester et al (2013) [70]	189		Overcoming Addictions +Smart Recovery meetings versus Smart Recovery meetings		Alcohol		3, 6 months		Abstinence, quantity, consequences	
	Hyland et al (2023) [71]	264		iCBT^m^+TAU versus TAU		Alcohol		3, 12 months		Abstinence, heavy use frequency, quantity, dependence severity, mental health symptoms, quality of life	
	Kelpin et al (2022) [39]	63		CBT4CBT+TAU versus residential TAU		Polydrug		12 weeks		Abstinence, use frequency	
	Kiluk et al (2016) [76]	68		CBT4CBT+TAU versus outpatient TAU		Alcohol		Posttreatment, 3, 6 months		Abstinence, heavy use frequency	
	Marsch et al (2014) [80]	160		TES+MMT versus MMT		Opioids		12 months		Abstinence	
	Paris et al (2018) [40]	92		CBT4CBT-Spanish+TAU versus outpatient TAU		Polydrug		Posttreatment, 3, 6 months		Abstinence	
	Schouten et al (2024) [44]	163		Beating the Booze+TAU versus TAU		Alcohol		3, 6 months		Quantity, dependence severity, mental health symptoms	
	Shi et al (2019) [89]	20		CBT4CBT+buprenophine+TAU versus buprenorphine maintenance+TAU		Opioids		0 weeks		Abstinence	
	Takano et al (2020) [112]	48		e-Learning Serigaya Methamphetamine Relapse Prevention Program+outpatient treatment versus self-monitoring+outpatient treatment		Polydrug		Posttreatment, 3, 6 months		Abstinence, cognitive outcomes	
	Tetrault et al (2020) [97]	58		CBT4CBT+standard care versus primary care standard care		Polydrug		0 weeks		Abstinence, use frequency	
**dCBI compared to CBI with a therapist**											
	Budney et al (2015) [55], arm 2	75		Computer-delivered MET/CBT/CM (COMPUTER) versus CBT+CM		Cannabis		Posttreatment, 9 months		Abstinence	
	Johansson et al (2020) [73]	301		Internet‐delivered CBT versus module content via therapy sessions		Alcohol		3, 6 months		Quantity, abstinence, peak consumption, dependence severity, mental health symptoms, quality of life	
	Kay-Lambkin et al (2009) [75], arm 2	97		Computer-delivered Self-Help for Alcohol and other drug use and Depression intensive therapy versus in-person CBT or MI^n^		Polydrug		3, 9 months		Heavy use frequency, mental health symptoms	
	Kay-Lambkin et al (2011) [74], arm 2	274		Clinician-assisted computerized treatment versus in-person CBT or MI		Polydrug		Posttreatment, 1, 4, 10 months		Severity, mental health symptoms, other	
	Kiluk et al (2018) [133], arm 2	137		CBT4CBT versus CBT in-person		Polydrug		Posttreatment, 6 months		Abstinence, dependence severity, frequency, consequences	
	Tiburcio et al (2018) [98], arm 1	83		Programa de Ayuda para Abuso de Drogas y Depresión versus CBT in-person		Polydrug		Posttreatment, 1 month		Use frequency, dependence severity, mental health symptoms	
	Tiburcio et al (2018) [98], arm 2	83		Programa de Ayuda para Abuso de Drogas y Depresión versus treatment center TAU with self-help guide		Polydrug		Posttreatment, 1 month		Use frequency, dependence severity, mental health symptoms	

^a^*K*=65 with 71 study arms.

^b^CBT: cognitive behavioral therapy.

^c^DBT: dialectical behavioral therapy.

^d^TAU: treatment as usual.

^e^MET: motivational enhancement therapy.

^f^CM: contingency management.

^g^CBT4CBT: computer-based training for cognitive behavioral therapy.

^h^TES: therapeutic enhancement system.

^i^MMT: methadone maintenance treatment.

^j^CRA: community reinforcement approach.

^k^ALC: alcohol (use).

^l^TXT: text messaging.

^m^iCBT: internet-based cognitive behavioral therapy.

^n^MI: motivational interviewing.

The primary substance targets were alcohol use (35/65, 53.85%), followed by polydrug use (13/65, 20%), with a majority of samples meeting criteria for an alcohol or substance use disorder (40/65, 61.54%). Further, 15.38% (10/65) of studies targeted a co-occurring condition, such as posttraumatic stress disorder [41] or depression [42]. While full details on study interventions can be found in Multimedia Appendix 2, the mean length of treatment was 12.20 (SD 15.17) modules or exercises, 36.92% (24/65) included some form of clinical support, such as online support [43] or feedback on homework [112], and over half of the interventions included elements of other evidence-based treatments (eg, motivational interviewing). For study context, participants were mostly recruited from the community via online advertising (30/65, 46.15%), followed by specialty mental health or addictions treatment facilities (17/65, 26.15%). A majority of studies took place outside of the United States (39/65, 60%). Finally, study-level risk of bias results showed that most studies had at least 1 high risk designation (which we then designated as high risk overall), and these were typically due to a lack of participant or personal blinding or incomplete outcome data (Multimedia Appendix 5).

### dCBI Effect Size by Outcome Class and Comparison Type

#### Overview

Primary study effect sizes were pooled by outcome class and comparison type. When effect sizes were pooled across all 4 between-condition comparisons, the overall pooled effect for consumption outcomes was small, positive, statistically significant, and heterogeneous (*g*=0.22, 95% CI 0.14-0.30; *P<*.001; τ=0*.*08; *I*^2^=76.7%; *k*=63; *k_es_*=274). Psychosocial outcomes showed a similar pattern of results (*g*=0.15, 95% CI 0.06-0.23; *P*=.001; τ^2^=0*.*06; *I*^2^=70.1%; *k*=39; *k_es_*=147). These findings were consistent across correlation values and similar to findings based on traditional meta-analytic methods (Multimedia Appendix 6). The following sections consider pooled effect sizes by each between-condition comparison, followed by within-condition comparisons.

#### dCBI Compared to Minimal Treatment

Studies of dCBI compared to assessment only or minimal treatment showed a small, positive, and significant effect for consumption (*g*=0.27, 95% CI 0.15-0.39; *P<*.001; τ^2^=0*.*10; *I*^2^=85.1%; *k*=31; *k_es_*=134) and psychosocial outcomes (*g*=0.16, 95% CI 0.05-0.27; *P*=.008; τ^2^=0*.*04; *I*^2^=75.2%; *k*=16; *k_es_*=60) over follow-up. Heterogeneity data showed systematic, relative to random variability, suggesting the utility of further moderation analyses. The 95% prediction interval suggested that future studies of dCBI for AOD with minimal treatment comparators may observe consumption effect sizes ranging from –0.39 to 0.93 and psychosocial effect sizes ranging from –0.29 to 0.61. Findings were consistent across correlation values and like those based on traditional meta-analytic methods (Multimedia Appendix 6). For assessment of bias due to publication status in studies contrasting dCBI with a minimal control, Figures S1 and S2 in Multimedia Appendix 7 show symmetry in the distribution of effect sizes by magnitude and SE. This does not suggest publication bias.

#### dCBI Compared to Another Treatment

Studies of dCBI compared to a control treatment or treatment as usual showed a nonsignificant effect for consumption outcomes over follow-up (*g*=0.08, 95% CI –0.23 to 0.39; *P*=.59; τ^2^=0*.*12; *I*^2^=80.0%; *k*=12; *k_es_*=53). Psychosocial outcomes were also nonsignificant (*g*=0.19, 95% CI –0.17 to 0.54; *P*=.26; τ^2^=0*.*15; *I*^2^=84.6%; *k*=10; *k_es_*=33). Heterogeneity data showed values above our threshold, but only the consumption pooled effect size met our sample size criterion. The 95% prediction interval suggested that future studies of dCBI for AOD compared to another treatment may observe consumption effect sizes ranging from –0.77 to 0.93 and psychosocial effect sizes ranging from –0.79 to 1.17. Findings were consistent across correlation values and like those based on traditional meta-analytic methods (Multimedia Appendix 6). For assessment of bias due to publication status in studies contrasting dCBI with another treatment, Figures S3 and S4 in Multimedia Appendix 7 do not suggest an association between SE and effect size.

#### dCBI as an Addition to Treatment as Usual

Studies of dCBI as an addition to treatment as usual, compared to treatment as usual alone, showed a small, positive, and significant effect for consumption outcomes over follow-up (*g*=0.23, 95% CI 0.14-0.31; *P<.*001; τ^2^*<.*01; *I*^2^=9.8%; *k*=20; *k_es_*=65), while psychosocial outcomes were nonsignificant (*g*=0.15, 95% CI –0.04 to 0.33; *P*=.11; τ^2^=0*.*06; *I*^2^=56.6%; *k*=12; *k_es_*=41). However, for psychosocial outcomes, Schouten et al [44] was an influential study, such that when removed, the effect size was statistically significant (*g*=0.15, 95% CI 0.02-0.29). Heterogeneity data for psychosocial outcomes were slightly above our threshold, and the 95% prediction interval was –0.43 to 0.73. Findings were consistent across correlation values and like those based on traditional meta-analytic methods (Multimedia Appendix 6). For assessment of bias due to publication status in studies testing dCBI as an addition to usual care, Figures S5 and S6 in Multimedia Appendix 7 do not suggest an association between SE and effect size.

#### dCBI Compared to CBI Delivered by a Therapist

A small number of studies in this review examined dCBI in comparison to CBI delivered by a therapist. These studies showed a nonsignificant effect for consumption (*g*=0.18, 95% CI –0.23 to 0.59; *P*=.27; τ^2^=0*.*11; *I*^2^=42.41%; *k*=5; *k_es_*=21) and psychosocial outcomes (*g<*0.01, 95% CI –0.24 to 0.24; *P*=.99; τ^2^=0*.*01; *I*^2^=25.2%; *k*=5; *k_es_*=15) over follow-up. Heterogeneity data did not suggest the need for further moderator analysis or calculation of prediction intervals. Findings were consistent across correlation weights and like those based on traditional meta-analytic methods (Multimedia Appendix 6). For assessment of bias due to publication status in studies testing dCBI compared to CBI delivered by a therapist, Figures S7 and S8 in Multimedia Appendix 7 do not suggest an association between SE and effect size.

#### dCBI Change From Baseline

While between-group effect sizes offer a measure of intervention effect compared to some form of control, within-group effect sizes provide information on the extent of change after receiving the intervention. For consumption outcomes over follow-up, the effect was large, positive, and statistically significant (*g*=0.71, 95% CI 0.62-0.80; *P<*.001; τ^2^=0*.*12; *I*^2^=92.6%; *k*=45; *k_es_*=201). Psychosocial outcomes were slightly smaller in magnitude, but the pattern of results was similar (*g*=0.52, 95% CI 0.39-0.66; *P<*.001; τ^2^=0*.*18; *I*^2^=94.4%; *k*=36; *k_es_*=131). Heterogeneity data suggested the utility of further moderation analyses. The 95% prediction interval suggests that future studies of dCBI for AOD may observe within-group consumption effect sizes ranging from 0.01 to 1.41 and psychosocial effect sizes ranging from –0.35 to 1.39. Findings were consistent across correlation weights and like those based on traditional meta-analytic methods (Multimedia Appendix 6).

### Moderation of Heterogeneous Effect Sizes

This study considered outcome class and comparison type as subgroup moderators to derive pooled effect sizes describing dCBI for AOD. However, significant residual heterogeneity was observed when dCBI was compared to minimal treatment (consumption and psychosocial outcomes), another treatment (consumption outcomes), when CBI was added to usual care and compared to usual care alone (psychosocial outcomes), and when within-dCBI effect sizes were estimated (consumption and psychosocial outcomes). In these sensitivity analyses, outcome time point (ie, posttreatment to 3 months vs 4 months or later) and primary drug targeted (ie, alcohol, polydrug, and other) were selected as study-level moderators in multivariate, correlated effects meta-regression models. For dCBI consumption outcomes in contrast to a minimal control, early follow-up studies were associated with smaller effect sizes than later follow-up studies, and polydrug studies showed smaller effects than alcohol studies. For dCBI psychosocial outcomes in contrast to a minimal control, polydrug studies also showed smaller effects than alcohol studies. The remaining pooled effect estimates, time point, and primary drug targeted were not significantly associated with effect size magnitude (see Table 3 for details).

**Table 3:** Moderation of heterogeneous pooled effect sizes.

Comparison			Consumption						Psychosocial				
			b (SE)		*df*		*P* value		b (SE)		*df*		*P* value
**dCBI^a^ compared to assessment only or minimal treatment^b^**													
	Estimate	*0.35 (0.08)^c^*		*18.81*		*<.001*		*0.21 (0.06)*		*8.63*		*.01*	
	Time point	–*0.18 (0.09)*		*20.27*		*.048*		0.02 (0.13)		7.86		.91	
	Polydrug	–*0.57 (0.08)*		*18.81*		*<.001*		–*0.21 (0.06)*		*8.63*		*.01*	
	Other	–0.05 (0.13)		9.17		.72		–0.11 (0.12)		6.94		.38	
**dCBI compared to another treatment or treatment as usual^d^**									—^e^		—		—
	Estimate	–0.29 (0.28)		5.63^f^		.34							
	Time point	0.27 (0.24)		8.10^f^		.30							
	Polydrug	0.85 (0.28)		1.66^f^		.12							
	Other	0.40 (0.29)		5.93^f^		.21							
**dCBI as an addition to treatment as usual^g^**													
	Estimate							0.22 (0.14)		5.66^f^		.17	
	Time point							–0.25 (0.12)		6.11^f^		.08	
	Polydrug							0.05 (0.20)		6.49^f^		.80	
	Other							–0.15 (0.22)		1.74^f^		.58	
**dCBI change from baseline^h^**													
	Estimate	*0.77 (0.06)*		*28.8*		*<.001*		*0.56 (0.11)*		*19.70*		*<.001*	
	Time point	–0.06 (0.07)		33.4		.43		–0.02 (0.10)		25.90		.86	
	Polydrug	—		—		—		0.15 (0.18)		10.30		.44	
	Other	–0.12 (0.11)		24.2		.27		–0.21 (0.14)		18.50		.15	

^a^dCBI: digitally delivered cognitive behaviorally based intervention.

^b^Consumption: *I*^2^=84.24, τ^2^=0.09; psychosocial: *I*^2^=70.34, τ^2^=0.05.

^c^Values in italics format indicate estimates significant at *P*<.05.

^d^Consumption: *I*^2^=78.70, τ^2^=0.13.

^e^Not applicable.

^f^Estimates <4 *df* unstable.

^g^Psychosocial: *I*^2^=60.36, τ^2^=0.08.

^h^Consumption: *I*^2^=92.49, τ^2^=0.12; psychosocial: *I*^2^=94.31, τ^2^=0.18.

### Pooled Effect Sizes for Each Outcome Type

**Figure 2:**
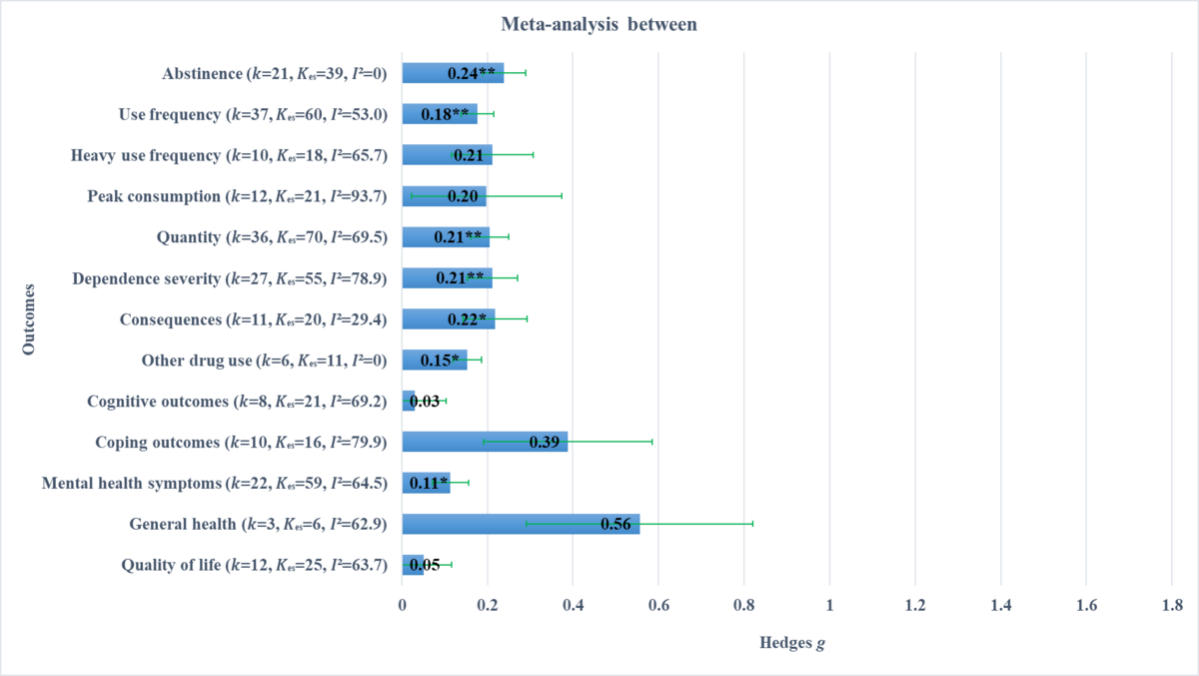
Between-group effect sizes (with SE) by outcome type. *P* values are marked to indicate statistical significance: *<.05, **<.01.

**Figure 3:**
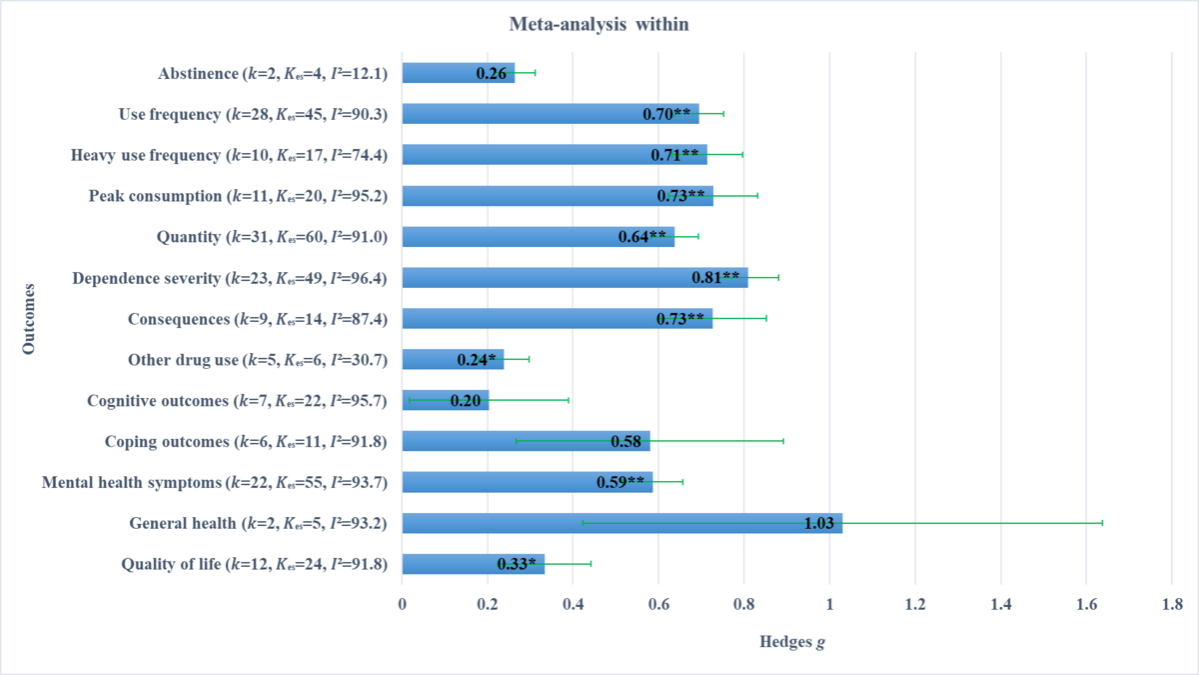
Within-group effect sizes (with SE) by outcome type. *P* values are marked to indicate statistical significance: *<.05, **<.01.

In this meta-analysis, 13 outcomes reported across 65 clinical trials of dCBI for AOD were aggregated into consumption and psychosocial outcome classes. In this final sensitivity analysis, we examined each outcome type individually to report pooled effect direction, magnitude, significance, and relative heterogeneity. The results of these analyses are separated by between- and within-group effect size types and are shown in Figures 2 and 3, respectively. Figure 2 shows between-group effects, where all pooled effect sizes were positive and mostly small in magnitude. Statistically significant pooled estimates were observed for abstinence, frequency, quantity, use severity, consequences, other drug use, and mental health, but not heavy frequency, peak consumption, cognitive outcomes, coping outcomes, general health, and quality of life. Estimates of between-study variance, relative to sampling error, showed that heterogeneity was still above moderate in most of these estimates. Figure 3 shows within-group effects, where most pooled effect sizes were positive and large in magnitude. Statistically significant pooled estimates were observed for frequency, heavy frequency, peak consumption, quantity, use severity, consequences, other drug use, mental health, and quality of life, but not abstinence, cognitive outcomes, coping outcomes, and general health. Estimates of heterogeneity were above moderate for most subgroup effect sizes.

## Discussion

### Overview

This is the first meta-analysis to evaluate the effect of dCBIs for AOD that included measures of psychosocial functioning as well as substance use consumption. The results generated here from 110 publications, involving 65 randomized controlled trials with over 24,000 combined participants, support the efficacy of dCBI at improving substance use and psychosocial outcomes with pooled findings suggesting a small relative benefit. However, more nuanced findings for between-group effects were apparent when studies were separated according to the type of comparison condition, with dCBI showing limited efficacy when compared to another treatment, in-person CBI, and when added to usual care (ie, nonsignificant efficacy in relation to psychosocial outcomes only). In total, 6 of 8 pooled between-group effect sizes showed statistical heterogeneity that was not fully accounted for by the time point of follow-up or the primary drug targeted within the clinical trial. Here, prediction intervals contained a wide range of consumption and psychosocial effect sizes that could be observed for dCBI for AOD in reference to minimal treatment, another treatment, or when added to usual care. Together, these sensitivity analyses suggest that there are unknown methodological or patient population factors driving variability in effects that were not identified by this study. Finally, within-condition effects that provide a nonexperimental estimate of change from baseline indicated significant, heterogeneous, and moderate to large effects for dCBI on consumption and psychosocial outcomes. These effects suggest that, on average, 60% to 80% of individuals who received dCBI showed improvement from baseline across various measures of substance use and functioning.

As is often the case for meta-analyses of behavioral therapies for substance use disorders [151], including CBT [6], there is little evidence of superior efficacy of dCBI when directly compared with another therapy. Significant and small between-group effects of dCBI were observed for both consumption and psychosocial outcomes when compared to assessment only or minimal treatment, with evidence suggesting larger effects in alcohol studies and for consumption outcomes, at earlier follow-up time points. Yet, dCBIs were not efficacious when compared to another treatment such as treatment as usual or CBI delivered by a therapist. This pattern is consistent with findings of digital mental health interventions more broadly [45,152], such that they appear generally effective at improving symptoms of psychological conditions but are not necessarily more effective than in-person psychotherapeutic interventions. In terms of comparative treatment benefit (ie, between-group effects), when dCBI was provided as an adjunct to treatment as usual, there were significant and small effects on consumption outcomes when compared to treatment as usual alone. However, this significant effect did not extend to psychosocial outcomes. It may be that the beneficial effects of adding dCBI to standard care are most relevant for improving AOD and not general life functioning. It should be noted that fewer studies in this between-group comparison reported psychosocial outcomes compared to consumption outcomes, and the magnitude of the effect size for this between-group comparison was similar to the effect size when all comparisons were combined, which was significant. Thus, sample size may have impacted the statistical inference on psychosocial outcomes for studies evaluating dCBIs as an adjunct to standard care. Furthermore, when one influential study [112] was removed from this analysis, the between-group effect reached statistical significance.

The findings with respect to substance use consumption outcomes are largely consistent with other meta-analytic examinations of dCBI (or related terminologies) for AOD. For instance, our prior meta-analysis of 15 published clinical trials of technology-delivered CBT for alcohol use found a significant and small effect as a stand-alone treatment in contrast to minimal treatment (*g*=0.20), but the effect was nonsignificant when compared to a more active control [14]. Most recently, Gregory et al [13] reported a small, statistically significant effect (*g*=–0.23) that favored dCBI in reducing substance use (alcohol, drugs, or alcohol or drugs combined) relative to controls in a meta-analysis of 18 published studies. Of note, the current meta-analysis is more comprehensive than existing reports both in terms of the number of studies included and the type and number of outcomes reported.

This is the first report of the pooled effect of dCBI on various psychosocial outcomes, including consequences of AOD, mental health symptoms, general health, quality of life, cognitive outcomes, and coping outcomes. This provides a more robust evaluation of dCBI efficacy and effects in terms of expanded definitions of recovery beyond alcohol or drug abstinence [15,153,154]. Although significant between-group effects demonstrating the efficacy of dCBI on psychosocial outcomes were more limited in scope as compared to consumption outcomes, it is notable that pooled between-group effect sizes for consequences and mental health outcomes were significant, as problems in these areas are often among the reasons for seeking treatment for AOD [155]. The within-group findings (ie, not compared to a control condition), while statistically heterogeneous, suggest that dCBI is effective at reducing other drug use and consequences from AOD, as well as improving mental health and quality of life. Thus, dCBI as an intervention can have a beneficial effect on some psychosocial outcomes, but the benefit across multiple domains may not be superior to that achieved through another established treatment for AOD.

### Strengths, Limitations, and Conclusions

This meta-analysis provides the most comprehensive view of dCBI outcomes to date. Therefore, a key strength of this work was the capacity to extract all available data from primary study reports and to report on 2 broad outcome classes—consumption and psychosocial—as well as 13 different outcome types. The extent of clinical effect data provided is substantial. However, there are some limitations to consider. For example, we prioritized published clinical trials to facilitate consistency in methodology, but it is unknown whether the inclusion of gray literature such as dissertations and conference abstracts would have changed our study findings. While our sample of studies is relatively large for a meta-analysis and certainly larger than dCBI reviews to date, sample size still may have impacted our capacity to observe statistical significance for some pooled effect estimates. Study reporting, however, is transparent as to the number of studies and effect sizes that each pooled effect represents. Reporting of demographic characteristics, especially race or ethnicity, was incomplete across studies, which restricts conclusions regarding generalizability. Limitations in sample sizes for certain outcomes also led to the grouping of measures such as motivation, craving, and self-efficacy into a single outcome type (ie, cognitive outcomes) when there are important conceptual differences between these measures. This limitation underscores the importance of future studies not only reporting all outcome data in descriptive format but also reporting all primary and secondary outcomes. As a result, more stable and informative pooled effect sizes would be possible in the future. It is also the case that some pooled estimates contained significant residual heterogeneity, which suggests that effect estimates were variable within groupings beyond what could be attributed to sampling error. While the primary drug targeted and the time point of follow-up were drivers in some studies, undiscovered moderators are possible. For instance, the varied characteristics of dCBIs included here (eg, range of dose and duration and integration with other evidence-based approaches) may have contributed to effect heterogeneity. The question of what defines CBT for AOD has become more challenging due to its evolution and diffusion over the years, resulting in new forms of CBT that may not fit traditional definitions but that have shared theoretical underpinnings, processes of change, and techniques [156]. Finally, the impact of user engagement on outcomes remains an unanswered question. Low engagement, or the lack of uptake and/or poor adherence, is one of the most widely cited challenges and is considered the greatest barrier to progress in digital mental health [157,158]. The lack of consistent reporting of rates of engagement with the dCBIs in the studies included here precluded an empirical investigation of this issue.

With these limitations in mind, the results of this meta-analysis show a modest benefit for dCBI as a stand-alone therapy and as an addition to usual care. Importantly, stand-alone effects were observed for consumption outcomes as well as measures of psychosocial functioning. Moreover, the comprehensive approach to outcome extraction resulted in effect estimates for a range of functional end points and suggests that across comparison conditions and length of follow-up, small effects can be observed on various consumption measures, including abstinence and use severity, and on psychosocial measures, such as consequences and mental health symptoms. When change from baseline is examined, the effects are moderate to large and statistically significant for all outcomes except for abstinence, cognitive and coping outcomes, as well as general health. Overall, the results of this meta-analysis provide further evidence supporting the beneficial effect, albeit small, of dCBIs in reducing substance use and suggest that the benefits may extend to some psychosocial outcomes. This is critical because recovery from AOD is not confined to substance use outcomes and should be considered holistically.

## Data Availability

The datasets generated or analyzed during this study are available from the corresponding author on reasonable request.

## Appendix

Multimedia Appendix 1 Sample search strategy.

Multimedia Appendix 2 Digitally delivered cognitive behaviorally based intervention and comparator descriptions.

Multimedia Appendix 3 Measures and outcome scoring.

Multimedia Appendix 4 PRISMA checklist.

Multimedia Appendix 5 Sample-level risk of bias assessment.

Multimedia Appendix 6 Meta-analysis with study effect sizes averaged to the study level.

Multimedia Appendix 7 Plot of assessment of publication bias.

## Abbreviations

AOD: alcohol and other drug use
CBI: cognitive behaviorally based intervention
CBT: cognitive behavioral therapy
dCBI: digitally delivered cognitive behaviorally based intervention
DSM: Diagnostic and Statistical Manual
PRISMA: Preferred Reporting Items for Systematic Reviews and Meta-Analyses
