# Supplementary material for: Digitally Delivered Cognitive Behavioral Interventions for Alcohol and Other Drug Use: Meta-Analysis Across Consumption and Psychosocial Outcomes
Source: JMIR Ment Health. 2026 May 19;13:e82370. doi: 10.2196/82370 (PMC13231115; doi:10.2196/82370)
Supplement: Multimedia Appendix 1 [file mental_v13i1e82370_app1.docx]

**MEDLINE (via PubMed):**

**RCT Filter:**

Lefebvre C, Glanville J, Briscoe S, Featherstone R, Littlewood A, Marshall C, Metzendorf M-I, Noel-Storr A, Paynter R, Rader T, Thomas J, Wieland LS. [Chapter 4: Searching for and selecting studies](https://www.google.com/url?q=https%3A%2F%2Feur01.safelinks.protection.outlook.com%2F%3Furl%3Dhttps%253A%252F%252Ftraining.cochrane.org%252Fhandbook%252Fcurrent%252Fchapter-04%26data%3D05%257C01%257Ccarol%2540lefebvreassociates.org%257Ccd4c04f9d76e4ccd75f408da4a4fa6bf%257Cfed02e5e8b814a3bb90b8d8d25150b3b%257C0%257C0%257C637904004716156015%257CUnknown%257CTWFpbGZsb3d8eyJWIjoiMC4wLjAwMDAiLCJQIjoiV2luMzIiLCJBTiI6Ik1haWwiLCJXVCI6Mn0%253D%257C1000%257C%257C%257C%26sdata%3DG3vDmkv35p2m07hCDiY9p%252FyREGVQeaz%252Bxu7S0rr%252F8Zg%253D%26reserved%3D0&sa=D&sntz=1&usg=AOvVaw0VTns3Np18qklSb08-C4V9). In: Higgins JPT, Thomas J, Chandler J, Cumpston M, Li T, Page MJ, Welch VA (editors). Cochrane Handbook for Systematic Reviews of Interventions version 6.3 (updated February 2022). Cochrane, 2022. Available from [www.training.cochrane.org/handbook](https://www.google.com/url?q=https%3A%2F%2Feur01.safelinks.protection.outlook.com%2F%3Furl%3Dhttps%253A%252F%252Ftraining.cochrane.org%252Fhandbook%26data%3D05%257C01%257Ccarol%2540lefebvreassociates.org%257Ccd4c04f9d76e4ccd75f408da4a4fa6bf%257Cfed02e5e8b814a3bb90b8d8d25150b3b%257C0%257C0%257C637904004716156015%257CUnknown%257CTWFpbGZsb3d8eyJWIjoiMC4wLjAwMDAiLCJQIjoiV2luMzIiLCJBTiI6Ik1haWwiLCJXVCI6Mn0%253D%257C1000%257C%257C%257C%26sdata%3DSNnXSuFKRuHi0%252Bq3RDssyWSBM7PNtpl7i9ZDa7leJQg%253D%26reserved%3D0&sa=D&sntz=1&usg=AOvVaw2b9PruV5mXC3cFjulJ13LV) [Ovid, PubMed]

- - [Launch](https://www.google.com/url?q=https%3A%2F%2Fwww.ncbi.nlm.nih.gov%2Fpubmed%3Fterm%3D%28%28%28randomized%2Bcontrolled%2Btrial%2B%5Bpt%5D%2BOR%2B%2522controlled%2Bclinical%2Btrial%2522%5BPublication%2BType%5D%2BOR%2B%2522randomized%2522%5BTitle%2FAbstract%5D%2BOR%2B%2522placebo%2522%5BTitle%2FAbstract%5D%29%2BOR%2B%28%2522clinical%2Btrials%2Bas%2Btopic%2522%2B%5Bmesh%3A%2Bnoexp%5D%29%2BOR%2B%28randomly%2B%5Btiab%5D%2BOR%2Btrial%2B%5Bti%5D%29%29%2BNOT%2B%28animals%2B%5Bmh%5D%2BNOT%2Bhumans%2B%5Bmh%5D%29%29&sa=D&sntz=1&usg=AOvVaw18ZgwG1V33A2YMs4PQSgVP) Cochrane RCT sensitivity and precision maximising filter for PubMed
  - From the ISSG Search Filters Resource

**Date Run: November 21, 2022**

**Results: 3,753**

**Search Strategy:**

(("Cognitive Behavioral Therapy"[MeSH Terms] OR "Secondary Prevention"[MeSH Terms] OR "Behavior Therapy"[MeSH Terms:noexp] OR "Behavior Therapy"[Text Word] OR "behaviour therapy"[Text Word] OR "behavioral therapy"[Text Word] OR "behavioral therapies"[Text Word] OR "behavioural therapy"[Text Word] OR "behavioural therapies"[Text Word] OR "CBI"[Text Word] OR "CBT"[Text Word] OR "cognitive therapy"[Text Word] OR "cognitive therapies"[Text Word] OR "cognitive behavioral"[Text Word] OR "cognitive behavioural"[Text Word] OR ("relapse prevention"[Text Word] OR "Secondary Prevention"[Text Word]) OR ("coping skills"[Text Word] OR "coping behavio*"[Text Word] OR "social skills training"[Text Word])) AND ("Substance-Related Disorders"[MeSH Terms] OR "Illicit Drugs"[MeSH Terms] OR "Designer Drugs"[MeSH Terms] OR "Heroin"[MeSH Terms] OR ("alcohol"[Text Word] OR "cocaine"[Text Word] OR "methamphetamine"[Text Word] OR "stimulant"[Text Word] OR "opiate"[Text Word] OR "opioid"[Text Word] OR "Heroin"[Text Word] OR "marijuana"[Text Word] OR "cannabis"[Text Word] OR "illicit drug*"[Text Word] OR "illegal drug*"[Text Word] OR "polysubstance"[Text Word])) AND (("randomized controlled trial"[Publication Type] OR "controlled clinical trial"[Publication Type] OR "randomized"[Title/Abstract] OR "placebo"[Title/Abstract] OR "clinical trials as topic"[MeSH Terms] OR ("randomly"[Title/Abstract] OR "trial"[Title])) NOT ("animals"[MeSH Terms] NOT "humans"[MeSH Terms]))) AND (1970/1/1: 3000/12/12[pdat])
