# Supplementary material for: Digitally Delivered Cognitive Behavioral Interventions for Alcohol and Other Drug Use: Meta-Analysis Across Consumption and Psychosocial Outcomes
Source: JMIR Ment Health. 2026 May 19;13:e82370. doi: 10.2196/82370 (PMC13231115; doi:10.2196/82370)
Supplement: Multimedia Appendix 2 [file mental_v13i1e82370_app2.docx]

**Table S1.** dCBI intervention and comparator descriptions.

| **First author (date)** | **Intervention description** | **Intervention dose (time to complete)^1^** | | **Flexibility (adherence)^2^** | | **Comparison (adherence)^3^** | |
| --- | --- | --- | --- | --- | --- | --- | --- |
| ***dCBI compared to assessment only or minimal treatment*** | | | | | | |  |
| Andersson (2015) [69] | **Internet intervention (WEB).** An email-based intervention targeting hazardous drinking by teaching PBS and offering personalized feedback based on participant alcohol use data. Modules were brief, with less than five-hundred words. They included information about pacing drinking, techniques to slow down consumption (e.g., drink water), the risk of alcohol tolerance, negative consequences associated with high BAC, and setting goals to reduce consumption. | 1-2 modules | | Participants were given 6 weeks to complete their sessions (85% completed all sessions assigned to them). | | Waitlist control group (86% completed follow-up). | |
|  |  |  | |  | |  | |
| Baumgartner et al (2021) [71] | **Adherence-focused Guidance Enhancement for Alcohol and Depression (AFGE-AD).** A 6-week online intervention based on CBT and MI designed to combat at risk alcohol use and depression symptomatology. The eight modules comprised 1) an introduction to the app, 2) setting and reaching goals, 3) positive activities and common problems, 4) the relationship between depression and common problems, 5) cravings, 6) slips, 7) meeting personal needs, and 8) preserving successes from the program. Additionally, adherence monitoring entailed tracking whether participants completed their weekly consumption diary, with reminders. | 8 modules | | Participants were told to complete 1-2 modules per week. Overall, they finished an average of 3.7 (SD = 2.8) modules. | | IAU (Internet as Usual) did not receive any treatment. | |
|  |  |  | |  | |  | |
| Baumgartner et al (2021) [72] | **CANreduce 2.0.** An online intervention based on CBT and MI in which fictional companions encourage participants to reflect on their cannabis use via educational modules. Topics addressed include identifying risky situations, addressing cravings, dealing with relapses, learning refusal skills, and building better personal habits. The intervention includes a daily use tracker, personalized email feedback, and an eCoach (automated virtual coach displayed before modules). | 8 modules | | Participants were given 6 weeks to complete the modules (M [SD] module completion was 2.6 [2.6]). | | Control participants were placed on a waiting list (11.2% completed the 6-month follow-up). | |
|  |  |  | |  | |  | |
| Blankers et al (2011) [75] comparison one | **Self-help alcohol online (SAO).** A standalone CBT and MI based online intervention that targets at risk alcohol use via modules with interactive feedback. The intervention includes four tiers. In the first, participants self-monitor their drinking and report on drinking-related cues; in the second, they are given personalized feedback on their drinking to compare their progress with their drinking goals; in the third, they are taught behavioral skills (e.g., coping with cravings); and in the fourth, they receive social support from other participants through an online forum. | 4 tiers | | Recommended that participants access program daily for at least 4 weeks (57% completed 6mo). | | Waitlist received no treatment (61% completed 6mo). | |
|  |  |  | |  | |  | |
| Bonar et al (2022) [76] | **Social media intervention.** A Facebook group intervention based on CBT and MI for cannabis use among emerging adults. Master's-level staff (e-coaches) posted content focused on dealing with stress, strategies to use safely, impact of cannabis on relationships, reducing use, and other topics. Content was presented to build rapport with participants online and be interactive, i.e., engaged participants could post or comment in response to advice from e-coaches. | 8 topics | | Participants could check the intervention as often as they wanted, as e-coaches posted about 6 posts per day (participant post/comment M [SD] was 28.1 [46.3]). | | Attention-matched control group that posted control content (e.g., memes/articles about animals/sports; participant post/comment M [SD] was 1.4 [3.6]). | |
|  |  |  | |  | |  | |
| Brendryen et al (2014)[77] | **Balance.** An interactive, computerized CBT intervention with 63 short sessions. The intervention has four foci 1) tracking alcohol use and commensurate goal setting, 2) relapse prevention, 3) emotion regulation, and 4) alcohol education. Sessions are supplemented by interactive tasks, quizzes, and homework. | 63 sessions  (3-10 min) | | The program releases sessions in a specific order (about one-fourth of participants completed 20 sessions or more). | | Brief screening session plus e-booklet with information about harms of alcohol use (79.0% of participants completed 6-month follow-up). | |
| Brief et al (2013) [52] | **VetChange.** An online intervention based on MI, CBT, and self-control for veterans with PTSD aiming to moderate or abstain from alcohol use. Eight modules that included how to set goals, evaluating risky situations, managing mood, and building support systems. The first three modules provided personalized feedback on drinking and PTSD symptoms; home exercises were provided throughout the intervention. | 8 modules  (20 min) | | Participants expected to complete one module per week (54% completed four or more modules). | | Waitlist control (61.2% completed first follow-up). | |
|  |  |  | |  | |  | |
| Chander et al (2021) [48] | **Computer delivered brief alcohol intervention (CBI).** A brief intervention based on a motivational and behavioral skills model. A 3-D avatar named Peedy the Parrot delivers a brief alcohol intervention with personalized feedback. Components of the intervention include decisional balance, identifying triggers and risky situations. | 1 session  (20 min) | | Intervention was given on the same day as baseline assessment (all participants completed intervention). | | Computer-delivered information on oral health (all participants completed intervention). | |
|  |  |  | |  | |  | |
| Cunningham (2012) [54] | **The Alcohol Help Center (AHC).** An online intervention based on CBT, MI, and relapse prevention. The exercises are divided into three sections focused on 1) how to initiate change, 2) key challenges entailed in trying to change drinking patterns, and 3) how to maintain these changes long-term. Additional components include a drinking diary, an online support group, and email/text messaging programs with encouragement and advice. | 20 exercises | | Participants could choose to do any of the exercises in any order (71.1% accessed intervention). | | Check Your Drinking is a brief, online personal feedback intervention (72.4% accessed intervention). | |
|  |  |  | |  | |  | |
| Daros et al (2024) [91] | **Pocket Skills 2.0.** A DBT-based online intervention for people with substance use disorders. Modules include distress tolerance, interpersonal effectiveness, and addiction skills. Each module presents a skill accompanied by a video displaying that skill; a practice session then occurs with a rule-based chatbot to help participants practice the skill they learned. | 5 modules | | Following completion of the introductory module, participants could access any modules they liked in any order for 12 weeks (M overall engagement 50 minutes). | | Delayed treatment group accessed intervention after four-week delay (M overall engagement 25 min). | |
|  |  |  | |  | |  | |
| Deady et al (2016) [53] | **DEAL Project.** An online intervention based on CBT and MI targeting comorbid alcohol use and depression. In four hour-long modules, participants were taught goal setting, behavioral activation, cognitive restructuring, problem solving, drink refusal, relapse planning, and other skills. Following modules, participants were given homework; homework results were reviewed when they began a new module. Participants were reminded via email to complete the modules. | 4 modules  (60 min) | | Participants completed one module per week (M [SD] sessions completed 1.50 [1.53]). | | Attention-matched control with four modules on improving overall well-being (M, [SD] sessions completed 2.50 [1.69]). | |
|  |  |  | |  | |  | |
| Gajecki et al (2017) [97] | **TeleCoach.** Web-based app designed to decrease excessive alcohol consumption. Participants log weekly alcohol consumption and receive personalized feedback. Also, depending on the user's preference, they are presented risky situation analysis and refusal skills (“say no to alcohol”) or relaxation techniques, positive thought exercises, and urge surfing skills (“feel better without alcohol”). | 2 main parts | | Participants were told to use the intervention at their discretion (80.6% available for analysis). | | Wait list control group (66.7% available for analysis). | |
|  |  |  | |  | |  | |
| Guillemont et al (2017) [101] | **Alcoometre.** A fully automated program based on principles of behavioral counseling to decrease alcohol misuse. The intervention consists of three steps. First, personalized normative feedback and advice on cutting down drinking; second, prompts to reflect on motivations for drinking and identify drinking habits; third, drinking diary and feedback that compares actual consumption to consumption goals. | 3 steps | | Participants could access the intervention whenever they wanted to (10% lost to follow-up after completing baseline). | | Informed consumption was hazardous and given information about hazardous drinking (18% lost to follow-up after completing baseline). | |
|  |  |  | |  | |  | |
| Hester and Delaney (1997) [46] | **Behavioral Self-Control Program for Windows (BSCPWIN).** A computer-based program drawing on behavioral self-control techniques. Participants were taught goal setting and self-monitoring, rate control and drink refusal, behavioral contracting with rewards, evaluating triggers, functional analysis, and relapse prevention. The program provides personalized normative feedback based on consumption data. | 8 modules  (15-45 min) | | Participants completed about one session per week; the program allowed them to preview future sessions and review completed ones (all participants completed the 10-week follow-up). | | Waitlist control (all participants completed the 10-week follow-up). | |
|  |  |  | |  | |  | |
| Johansson et al (2021) [108] | **Self-help internet-based CBT (ICBT).** A computer/phone intervention for alcohol use based on MI, relapse prevention, and behavioral self-control. Modules were videos with skill examples or interviews with experts, and users were prompted to relate the content to their own lives. Participants were given access to a drinking calendar through which they could track their use; the program used these data to generate personalized feedback. | 5 main modules | | All modules were available to participants at the outset (main module completion ranged from 8.6% to 67.6%). | | Text-only information on changing alcohol use (47.4% completed 6-month follow-up). | |
|  |  |  | |  | |  | |
| Kramer et al (2009) [123] | **Drinking Less TV self-help course.** A television-based CBT intervention to reduce alcohol consumption. The episodes depict an addiction coach and two participants; the coach advises the participants on how to decrease their drinking and provides feedback on the progress they made since the prior episode. Four stages of content are presented 1) evaluating consumption levels, 2) goal setting, 3) preparing for risky situations, and 4) relapse prevention. The television program is supplemented by a corresponding website which includes an alcohol diary, graphical feedback, and a user forum. | 5 sessions  (25 min) | | Participants were sent one session per week via DVD (63.4% of participants viewed 4-5 sessions). | | Waitlist control group (96.7% completed the posttest). | |
|  |  |  | |  | |  | |
| Leeman et al (2016) [124] | **Tertiary Health Research Intervention via Email (THRIVE).** A brief, single-session intervention designed to teach cognitive-behavioral PBS to college students. Upon inputting drinking information, the program produces personalized feedback regarding dependence risk, estimated amount of money spent on alcohol, estimated peak 30-day BAC, and drinking patterns relative to age/sex norms. The program presents nine strategies that are either direct (e.g., alternate between alcohol and water) or indirect (e.g., have friends support safe drinking goals). | 1 session  (9 min) | | Participants completed the program in one sitting (84% received intervention). | | Short electronic brochure (92.3% received intervention). | |
|  |  |  | |  | |  | |
| Liang et al (2018) [58] | **S-Health.** A mobile phone intervention based on CBT designed to help patients identify triggers, recognize strategies for dealing with triggers, and monitor substance use and cope with cravings. Participants received text messages daily, prompting them to self-monitor via completing surveys on triggers, cravings, and affect. | Daily for 28 days | | Participants were asked to take daily surveys at any time each day, and they could take multiple in one day (94% of participants retained). | | Text messages with psychoeducation (none of the participants discontinued the intervention). | |
|  |  |  | |  | |  | |
| Mujcic et al (2022) [130] | **MyCourse.** An alcohol moderation intervention based on MI, CBT, and ACT for cancer survivors. Using a phone, computer, or tablet, participants can access the intervention to set a quit or moderation date, log alcohol use and cues, access a peer support forum, and practice skills via interactive exercises. | Daily for 28 days | | Participants were encouraged to log in daily for four weeks (median times accessed course = 8). | | Non-interactive online informational pamphlet (68% completed 12-month follow-up). | |
|  |  |  | |  | |  | |
| Riper et al (2008) [136] | **Drinking Less (DL).** An online intervention based on CBT and self-control principles for individuals looking to reduce alcohol use to low-risk consumption. Four successive stages of intervention 1) preparing for action, 2) goal setting, 3) behavior change, and 4) maintenance of gains and relapse prevention. A moderated discussion forum was also made available. | 4 stages | | Participants allowed to use intervention as much as they liked (45.4% of participants used DL). | | Web-based psychoeducational brochure (51.1% of participants used the brochure). | |
|  |  |  | |  | |  | |
| Schaub et al (2015) [141] | **Can Reduce.** A web-based intervention based on CBT and MI designed to reduce cannabis use. Thirteen modules taught participants how to moderate their use; examples include identifying risk situations, dealing with cravings and relapses, and refusing cannabis. The program also included a consumption diary and a knowledge base with harm reduction techniques and information about the consequences of cannabis use. | 8 modules | | Participants were told to complete the modules in a specific order (participants completed on average about three modules). | | Waitlist control (59.1% completed 3-month follow-up). | |
|  |  |  | |  | |  | |
| Schaub et al (2019) [140] | **Snow Control.** An online intervention based on CBT and other empirically supported therapies for cocaine use. Participants could choose one of six virtual companions to guide them through eight psychoeducation modules, which covered risky situations, cravings, slips, and saying no to cocaine. The intervention also included a consumption diary in which participants inputted their goals and their actual use; graphical feedback displayed the extent to which the two converged and gave participants words of encouragement. | 8 modules | | Participants encouraged to complete 1-2 modules per week in the order they were presented (M [SD] modules completed 2.71 [2.44]). | | Waitlist control (17.9% completed 6-month follow-up). | |
|  |  |  | |  | |  | |
| Sinadinovic et al (2014) [146]  comparison one | **Alkoholhjalpen.se.** An online intervention based on CBT and MI for problematic alcohol use. Eighteen modules cover topics such as risky situations, consequences, progress rating, and decisional balance, with videos and interactive exercises. Participant responses yield personalized recommendations from the program about which modules to pursue. Also, an electronic diary and a discussion forum are available for use. | 18 modules | | Participants could choose whichever modules they wanted to work on in any order (53.8% accessed intervention). | | Waitlist control group (47.6% completed 12-month follow-up). | |
|  |  |  | |  | |  | |
| Sinadinovic et al (2020) [145] | **A way out of fog.** An online intervention for cannabis users based on CBT and MI. Thirteen modules covered topics such as motivation to change, relapse prevention, managing cravings, and coping with difficult emotions. Content was presented as text (with optional voice-over) followed by questions. Additionally, users had access to a use calendar, which produced personalized feedback, and a therapist with whom they could correspond via text. | 13 modules | | Recommended to complete 1-2 modules per week (M [SD] modules completed 3.9 [2.7]). | | Waitlist control group (48.7% completed follow-up). | |
|  |  |  | |  | |  | |
| Stapinski et al (2021) [147] | **Inroads anxiety and alcohol use intervention.** A web-based CBT intervention tailored for emerging adults with anxiety and alcohol use problems. Modules include topics understanding patterns of alcohol use, the relationship between anxiety and drinking, CBT strategies for anxious thoughts, CBT strategies for alcohol expectancies and sticking to limits, relapse prevention, and avoidance and anxiety. Engagement was optimized via videos, vignettes, and quizzes. Telephone sessions with a clinical psychologist were offered if participants wanted additional support. | 5 modules | | Each week, one new module became accessible (more than a third of the sample completed all modules). | | Assessment with alcohol information (77% completed 6-month follow-up). | |
|  |  |  | |  | |  | |
| Sunami et al (2022) [148] | **Sensible and Natural Alcoholism Prevention Program for You: Diary On Computer (SNAPPY-DOC).** An online intervention for problem drinking based on self-monitoring. Participants set goals for their drinking, logged their use daily, and received visual feedback based on their progress. CBT/MI-derived feedback comments encouraged participants to reflect on their habits. Participants also received psychoeducation on costs/benefits of alcohol, drinking triggers, and improvements due to drinking less. | Daily for 28 days | | Participants were told to use the program daily (all participants completed follow-ups). | | Assessment only control (all participants completed follow-ups). | |
|  |  |  | |  | |  | |
| Sundstrom et al (2020) [149] | **Low-intensity internet intervention.** A CBT-based intervention for alcohol use disorder. Nine modules include setting goals, managing risky situations, preparing a relapse plan, dealing with urges, and refusing alcohol in social situations. Each module is accompanied by a homework exercise. Participants are notified by text when a new module is available. | 9 modules | | New modules continually made available throughout course of experiment (M [SD] modules completed 5.9 [2.8]). | | Waitlist control (95.7% completed post-treatment assessment). | |
|  |  |  | |  | |  | |
| Tait et al (2014) [150] | **breakingtheice.** A web-based intervention for amphetamine users based on MI and CBT. In line with harm reduction, participants are instructed to choose a goal (e.g., complete abstinence, or moderation, or use more safely) and are presented with three modules. The first covers the connection between use and life problems (e.g., friendship, employment); the second covers the pros and cons of use and trade-offs of behavior change; and the third covers behavior change techniques (e.g., setting goals, overcoming cravings). | 3 modules  (30 min) | | One module per week was recommended but participants were allowed to proceed at their own pace (48% completed all three modules). | | Waitlist control (52% completed 6-month follow-up). | |
|  |  |  | |  | |  | |
| Wallace et al (2011) [47] | **Down Your Drink (DYD).** An online intervention for hazardous alcohol use based on MET, CBT, behavioral self-control, and relapse prevention. Participants were presented with three phases: decision making, implementing change, and relapse prevention. Phases were supplemented by exercise and tasks, as well as e-tools (e.g., daily diary for drinking and triggers and emotions). | 3 phases | | Participants could complete the modules as often as they liked (M [SD] number of visits to site 2.33 [3.63]). | | Information about negative consequences of excessive drinking without other site components (M [SD] number of visits to site 1.24 [0.75]). | |
|  |  |  | |  | |  | |
| Wilks et al (2018) [156] | **Internet-delivered DBT skills training intervention (iDBT-ST).** A tech intervention based on DBT for suicidal and heavy episodic drinking. Eight modules focused on four themes 1) mindfulness, 2) reducing problematic drinking, 3) emotion regulation, and 4) distress tolerance. Each module included a video segment followed by a key point summary and guided practice. Participants also select a homework task and a worksheet would be emailed to them; email/text reminders ensured that participants stayed engaged. | 8 modules  (30-50 min) | | Modules released over the course of 8 weeks (throughout the study, the number of people who attempted the next session declined by an average of about 5 per subsequent session). | | Waitlist control (96.6% completed 2-month follow-up). | |
|  |  |  | |  | |  | |
| Zill et al (2019) [158] | **Vorvida.** A phone/computer intervention based on CBT and MI for at risk alcohol use. Four CBT modules target 1) motivations, consequences, and goals, 2) cravings and triggers, 3) risky situations, and 4) slips and relapses. Content can be presented as text or audio. As participants interact with the program, it adapts to the individual and tailors responses using rules-based artificial intelligence. The intervention also includes a mood and alcohol tracker and daily text reminders. | 4 modules | | Users can engage with the program at their own pace, but two hours per week is recommended (62.7% completed t2 follow-up). | | Waitlist control (77.2% completed t2 follow-up). | |
|  |  |  | |  | |  | |
| ***dCBI compared to another treatment or treatment as usual*** | |  |  | |  | |  |
| Augsburger et al (2022) [70] | **On-line self-help intervention.** An online intervention based on CBT and MI for alcohol misuse. Ten video modules taught participants about behavioral skills such as self-monitoring, setting goals, refusing drinks, risky situations, and coping with cravings. The program included a consumption diary for tracking and visualizing weekly use. Additionally, an e-coach sent automated encouragement and reminder messages to participants. | 10 modules | | The order of the modules was fixed, but participants could skip/review modules (median completed modules = 3). | | Interactive psychoeducation website with self-test and tailored feedback (73.1% completed follow-up). | |
|  |  |  | |  | |  | |
| Berman et al (2020) [74] | **TeleCoach**. A smartphone-based skills training app designed to reduce risky alcohol use. Participants were taught to self-monitor (using a drinking calendar), practice relapse prevention skills (such as saying "no" to alcohol), and deploy emotion regulation techniques (such as urge surfing). | 3 components, participants used app for about 6.5 minutes when accessed | | Participants were given access to the intervention for one month (M [SD] app visits 3.56 [4.13]). | | Control app offered brief advice regarding problematic alcohol use (M [SD] app visits 4.39 [4.89]). | |
| Blankers et al (2011), [75] comparison two | **Self-help alcohol online (SAO).** A CBT and MI based online intervention that targets at risk alcohol use via modules with interactive feedback. The intervention includes four tiers. In the first, participants self-monitor their drinking and report on drinking-related cues; in the second, they are given personalized feedback on their drinking to compare their progress with their drinking goals; in the third, they are taught behavioral skills (e.g., relapse prevention, coping with cravings); and in the fourth, they receive social support from other participants via an online forum. | 4 tiers | | Recommended that participants access program daily for at least 4 weeks (57% completed 6mo). | | Therapy Alcohol Online received text-based therapy based on the CBT/MI protocol (60% completed 6mo). | |
|  |  |  | |  | |  | |
| Budney et al (2015), [79] comparison one | **Computer-delivered MET/CBT/CM (COMPUTER).** A CBT/MET intervention for individuals with cannabis use disorder. Nine online modules were adapted from TES, which is derived from CRA and CBT principles. Themes included strategies for drug refusal, working with thoughts about drug use, and functional analysis. Treatment was supplemented with three supportive counseling sessions and a CM program. | 12 sessions  (9 computer modules, 3 counseling sessions) | | Participants completed modules at the site with staff nearby for technical support (M [SD] computer/counseling sessions 4.8 [3.4]). | | Two brief counseling sessions (M [SD] sessions 1.4 [0.5]). | |
|  |  |  | |  | |  | |
| Gonzales and Dulin (2015) [99] | **Location-Based Monitoring and Intervention for Alcohol Use Disorders (LBMI-A).** A cell phone-based cognitive-behavioral intervention with seven modules, teaching skills such as managing cravings, solving problems, adopting alternative nondrinking activities, and identifying risky settings that could prompt use. The program enabled participants to use coping strategies in real-time and monitor their drinking. The program also generated weekly feedback based on user data. | 7 steps | | Participants checked the app on their phone at their discretion (71.4% launched all of the system steps at least once). | | Brief online motivational intervention plus psychoeducation booklet (participants read an average of 8 pages of the online booklet). | |
|  |  |  | |  | |  | |
| Kay-Lambkin et al (2009) [113] comparison one | **Computer-delivered Self-Help for Alcohol and other drug use and Depression (SHADE) intensive therapy.** An online intervention based on CBT and MI for comorbid depression and alcohol/cannabis use disorder. In line with harm reduction, participants choose their therapy goals; content helped them explore the relationship between their substance use and their depression. Participants are shown video demonstrations, given exercises to learn CBT skills, and meet with a therapist post-session to review progress and schedule the next week’s appointment. | 9 sessions  (12 min) | | Participants told to complete nine sessions in sequence once a week (M [SD] sessions attended 7.61 [2.87]). | | Brief intervention alone followed by no treatment. | |
|  |  |  | |  | |  | |
| Kay-Lambkin et al (2011) [112] comparison two | **Clinician-assisted computerized [CAC] treatment.** An online intervention with minimal therapist guidance based on CBT and MI for comorbid depression and substance use. Participants completed computerized sessions followed by brief ten-minute individual check-ins with a trained psychotherapist. The first of nine sessions, for example, covered assessment feedback, psychoeducation about substance use and depression, and initial goal setting. | 9 sessions  (60 min allotted) | | Participants attended sessions once per week (29.9% received all sessions). | | Nine weekly hour-long sessions of person-centered therapy (30.3% received all sessions). | |
|  |  |  | |  | |  | |
| Kiluk et al (2018), [119] comparison one | **CBT4CBT.** An online intervention that includes voice-over narration, animations, quizzes, and other interactive elements to help teach CBT skills. Use of the program was supplemented by brief in-person check-ins with clinicians. Skills taught include 1) understanding use patterns, 2) coping with cravings, 3) refusing substances, 4) developing problem-solving skills, 5) changing thinking about substances, and 6) improving decision-making skills. | 7 modules | | Participants were asked to complete one module per week (M [SD] of modules was 5.5 [2.3]). | | TAU (12 group sessions offered with individual as-needed, M [SD] 5.6 [3.1]). | |
|  |  |  | |  | |  | |
| O’Donnell et al (2019) [133] | **Minimise.** An ecological momentary intervention based on PBS for young adult alcohol use. Participants set goals to reduce use or reduce consequences. Over 28 days, participants were sent two notifications per day to complete self-monitoring surveys, in which they reported intention to use, use levels, consequences, social context, affect, and PBS. In real time they were given feedback to review strategies related to their reported goals, affect, and social context. | 2 reminders for 28 days | | Participants sent notifications twice daily (M [SD] number of days engaged with app 22.08 [9.70]). | | Self-monitoring with daily prompts to enter and view alcohol consumption and context data (M [SD] number of days engaged with app 22.1 [8.55]). | |
|  |  |  | |  | |  | |
| Olthof et al (2023) [134] | **ICan**. A smartphone app targeting moderate cannabis use based on CBT and MI. The intervention begins with a brief screening plus personalized feedback, then helps participants create a plan for decreasing use. To support this plan, modules teach goal setting, support seeking, and other skills. The intervention also includes a peer support platform and a cannabis diary. | 6 modules  (5 required, 1 optional) | | Participants follow prespecified module flow (M [SD] modules completed 2.8 (2.5). | | Online educational modules about reducing cannabis use (35.3% viewed all modules). | |
|  |  |  | |  | |  | |
| Rooke et al (2013) [137] | **Reduce Your Use:** How to Break the Cannabis Habit. A web-based intervention targeting problematic cannabis use based on CBT and MI. Module content includes changing your thinking, relapse prevention, and coping strategies. Exercises, as well as a cannabis diary, supplement the module content. | 6 modules | | Modules completed sequentially at participant's pace (M modules completed 3.5). | | Six control sections contain information about cannabis but not about building skills (52% completed 3-month follow-up). | |
|  |  |  | |  | |  | |
| Schaub et al (2012) [142] | **Snow Control.** An online intervention targeting cocaine addiction based on CBT, MI, self-control, and relapse prevention. Participants received 8 main modules, 4 additional modules, and a consumption diary. | 8 modules | | New modules were made accessible each week (M [SD] modules completed 2.60 [2.04]). | | Eight-module online psychoeducational control (M [SD] modules completed 1.80 [1.60]). | |
|  |  |  | |  | |  | |
| Sinadinovic et al (2014) [146] comparison two | **Alkoholhjalpen.se.** An online intervention based on CBT and MI for problematic alcohol use. Eighteen modules cover topics such as risky situations, consequences, progress rating, and decisional balance, with videos and interactive exercises. Participant responses yield personalized recommendations from the program about which modules to pursue. Also, an electronic diary and a discussion forum are available for use. | 18 modules | | Participants could choose whichever modules they wanted to work on in any order (53.8% accessed intervention). | | Online brief screening, personalized feedback, and motivational intervention (40.8% completed 12-month follow-up). | |
|  |  |  | |  | |  | |
| ***dCBI plus TAU versus TAU only*** | |  | |  | |  | |
| Acosta et al (2017) [67] | **Thinking Forward + TAU.** A self-directed computerized intervention based on CBT that targets heavy drinking among veterans with substance use disorder and PTSD. Twenty-four modules taught CBT skills such as identifying, evaluating and challenging thoughts and doing a functional analysis of alcohol or other drug use. Graphs displayed progress in reducing symptoms based on user input. | 24 modules  (20 min) | | Participants were told to complete two modules per week for the first six weeks and to review completed modules or complete optional modules thereafter (M [SD] modules completed 8.8 [6.2]). | | TAU comprised typical Veteran’s Administration services (e.g., social work, medical, behavioral, pharmacological; 91.4% received allocated intervention). | |
|  |  |  | |  | |  | |
| Budney et al (2015) [79] comparison two | **Computer-delivered MET/CBT/CM (COMPUTER).** A CBT/MET intervention for individuals with cannabis use disorder. Nine online modules were adapted from TES, which is derived from CRA and CBT principles. Themes included strategies for drug refusal, working with thoughts about drug use, and functional analysis. Treatment was supplemented with three supportive counseling sessions and a CM program. | 12 sessions  (9 computer modules, 3 counseling sessions) | | Participants completed modules at the site with staff nearby for technical support (M [SD] computer/counseling sessions 4.8 [3.4]). | | Individual counseling plus CM (M [SD] sessions 5.3 [3.4]). | |
|  |  |  | |  | |  | |
| Campbell et al (2014) [80] | **TES + TAU.** A 12-week computer-based treatment CM and 62 online modules based on CRA. Modules teach basic CBT and psychosocial functioning skills, as well as sexually transmitted infection prevention. | 62 modules  (20-30 min) | | Patients completed an average of 36.6 modules (SD = 18.1) out of a recommended 48. | | TAU. Relative to TES, TAU participants completed similar numbers of therapy sessions at their treatment centers. | |
|  |  |  | |  | |  | |
| Campbell et al (2023) [82] | **Therapeutic Education System-Native Version (TES-NAV) + TAU.** An app-based intervention to decrease substance use based on CRA and CM, tailored to the unique needs of Native American adults. Module themes include drug refusal, functional analysis, and working with thoughts about drug use. Supplemented by outpatient TAU. | 26 modules | | Participants asked to use intervention twice weekly and complete four modules per week (M [SD] modules completed 22.8 [24.3]). | | TAU included group therapy, drug education, individual counseling, and cultural activities (M [SD] hours of treatment 33.6 [32.1]). | |
|  |  |  | |  | |  | |
| Carroll et al (2008) [84] | **CBT4CBT + TAU.** A computer-based CBT-derived intervention for substance dependence. Six modules teach 1) understanding use patterns, 2) coping with cravings, 3) refusing substances, 4) developing problem-solving skills, 5) changing thinking about substances, and 6) improving decision-making skills. Skills are taught through video vignettes, in which actors navigate risky situations and model CBT skills; participants can interact with these vignettes and change their trajectory. Interactive assessments and homework are included in the program. | 6 modules  (45 min) | | Following module one, participants could access the remaining modules in any order and review/repeat those they completed (M number of computer sessions 4.3). | | TAU involved standard treatment including counseling (M 4.5 individual and 6.7 group sessions completed). | |
|  |  |  | |  | |  | |
| Carroll et al (2014) [87] | **CBT4CBT + TAU.** An online, CBT-based intervention for people with cocaine dependence on MMT. Seven modules teach 1) understanding use patterns, 2) coping with cravings, 3) refusing substances, 4) developing problem-solving skills, 5) changing thinking about substances, 6) improving decision-making skills, and 7) HIV risk reduction. Skills are taught through video vignettes, in which actors navigate risky situations and model CBT skills; participants can interact with these vignettes and change their trajectory. Interactive assessments and homework are included in the program. | 7 modules  (35 min) | | Participants were given a private room and computer on which to complete modules (M [SD] computer sessions completed 5.1 [2.3]). | | Standard treatment, namely MMT and counseling sessions (M 4.2 individual and 5.2 group sessions). | |
|  |  |  | |  | |  | |
| Carroll et al (2018) [88] | **CBT4CBT + TAU.** An online intervention that uses vignettes, interactive exercises, and quizzes to teach patients CBT skills that will enable them to decrease their substance use. Skills taught include 1) understanding use patterns, 2) coping with cravings, 3) refusing substances, 4) developing problem-solving skills, 5) changing thinking about substances, and 6) improving decision-making skills. | 7 modules  (30-40 min) | | Participants completed modules in private on a weekly basis (on average they completed 5-7 modules). | | TAU included MMT and weekly counseling sessions (average of 3 group and 7 individual). | |
|  |  |  | |  | |  | |
| Christensen et al (2014) [89] | **CRA + CM (CRA+).** An online intervention based on CRA; module topics include self-management and drug-refusal. Participants watched videos featuring actors in risky situations and had the ability to determine the story's progression—they received feedback on the soundness of their choices relative to the intervention’s advice. Module presentation was affected by how quickly and correctly participants responded to quiz questions, and personalized worksheets were generated post-session. | 69 topics  (30 min) | | Participants completed modules three times per week in a personalized order recommended by a therapist (80.4% treatment completion). | | CM and buprenorphine (64.1% treatment completion). | |
|  |  |  | |  | |  | |
| Farren et al (2015) [96] | **Cognitive relapse prevention-based computerised therapy + TAU.** A computer-based relapse prevention intervention modeled on Project MATCH. Participants completed five sessions focused on thoughts, feelings, and behaviors related to alcohol relapses with case histories and interactive questions. Session topics included countering inaccurate thoughts about alcohol use disorder, working with distressing feelings surrounding use, preventing relapse, and learning to reduce cravings. Participants received personalized feedback upon each session’s completion. | 5 sessions  (50 min) | | Participants left in a room alone to complete the sessions (64.5% of participants neither withdrew nor failed to complete protocol). | | Attention-matched control (simple mental math problems) and TAU with individual psychotherapy, self-help meetings, and psychiatric evaluations (62.5% of participants neither withdrew nor failed to complete protocol). | |
|  |  |  | |  | |  | |
| Glasner et al (2020) [98] | **Text-messaging CBT intervention (ALC-TXT-CBT).** One CBT session followed by a text message intervention based on CBT to target alcohol use and antiretroviral medication adherence. Targeted coping skills changed weekly. They included goal setting, managing negative emotions, relapse prevention, lifestyle choices, and identifying triggers. Some messages were static; others were interactive, prompting the user to respond. | 12 thematic emphases | | Participants received daily text messages for 12 weeks (89% received intervention). | | HIV TAU plus informational pamphlet on alcohol use and medication adherence (82% received intervention). | |
|  |  |  | |  | |  | |
| Guarino et al (2018) [100] | **Take Charge of Pain + TAU.** CBT-based program targeting coping with pain and “aberrant drug behavior.” Modules teach effective coping strategies such as pacing activity, challenging automatic thoughts, breath control, and muscle relaxation; the program also includes education about medication misuse and opioids. Interactive features include an activity calendar, a pain tracker, and a voice-over option. | 27 modules  (20-30 min) | | It was recommended that participants complete two modules per week (M [SD] modules completed 19 [10]). | | Opioid therapy TAU (all participants received allocated intervention). | |
|  |  |  | |  | |  | |
| Hester et al (2011) [103] | **ModerateDrinking.com + Moderation Management.** ModerateDrinking.com is an online intervention based on behavioral self-control training for problem drinking. It provides structured, interactive, individualized content in the form of modules addressing motivation, identifying and managing triggers, problem solving, and dealing with relapse. Participants set goals and monitor progress over time. Participants were also given access to Moderation Management. | 21 modules | | There is a recommended order, but participants can choose to complete modules that best suit their needs (average number of modules tapped = 8.9). | | Moderation Management alone, an online mutual help group for drinkers that offers meetings and psychoeducation (76.2% completed 12-month follow-up). | |
|  |  |  | |  | |  | |
| Hester et al (2013) [105] | **Overcoming Addictions (OA) + Smart Recovery meetings.** A web-based intervention based on the CBT-derived principles of Smart Recovery to help individuals stop drinking. The site contains modules, exercises, and graphic feedback, personalized to the needs of the user. Five modules are presented 1) Getting Started, 2) Building and Maintaining Motivation for Change, 3) Dealing with Urges and Cravings, 4) Self-Managing Thoughts, Feelings and Behaviors, and 5) Lifestyle Balance for Preventing Relapse. | 5 modules | | Program allows participants to complete modules in order they prefer (73.5% completed 6mo follow-up). | | Smart Recovery meetings (70.9% completed 6mo follow-up). | |
|  |  |  | |  | |  | |
| Hyland et al (2023) [106] | **iCBT + TAU.** An online intervention for people with alcohol dependence based on multiple approaches including CBT. Module content included relapse prevention, problem solving, and self-control strategies. Treatment was supplemented by primary care TAU. | 8 modules  (5 main, 3 optional) | | Open-ended, meaning participants could access any modules as often as they liked (M assignments completed 4.67). | | TAU entailed primary care for alcohol-dependent patients (70% completion of 12-month follow-up). | |
|  |  |  | |  | |  | |
| Kelpin et al (2022) [49] | **CBT4CBT + TAU.** A computerized CBT treatment program with seven modules, covering core CBT topics. Modules portray actors in risky situations employing CBT skills; videos were supplemented with homework and interactive exercise. Skills taught include 1) understanding use patterns, 2) coping with cravings, 3) refusing substances, 4) developing problem-solving skills, 5) changing thinking about substances, and 6) improving decision-making skills. | 7 modules  (M = 31 min) | | Women in the residential treatment program were given blocked-out time to complete the modules (M [SD] modules completed 5 [2]). | | TAU entailed group counseling, medication management, and case management (65.5% completed 12-week follow-up). | |
|  |  |  | |  | |  | |
| Kiluk et al (2016) [116] | **CBT4CBT + TAU.** A computer-based intervention with modules designed to mimic in-person CBT sessions. The program includes introductions to skills, didactic instructions, and opportunities to practice CBT skills. Skills taught include 1) understanding use patterns, 2) coping with cravings, 3) refusing substances, 4) developing problem-solving skills, 5) changing thinking about substances, and 6) improving decision-making skills. | 7 modules  (45 min) | | Participants were told to complete one module per week (M [SD] modules completed 5.6 [1.9]). | | Group or individual weekly psychotherapy sessions (M [SD] sessions attended 4.3 [2.2]). | |
|  |  |  | |  | |  | |
| Marsch et al (2014) [127] | **TES + MMT.** Participants completed a half hour of counseling and a half hour of the TES program. TES is derived from CRA and CBT principles. Module themes include strategies for drug refusal, working with thoughts about drug use, and functional analysis. Non-core modules were included if deemed necessary based a on a participant risk assessment survey. | 53 core modules  (15 min) | | Participants completed modules once per week for the first four weeks and twice monthly thereafter; participants given thirty minutes to complete about two modules (M [SD] modules completed 28 [24]). | | MMT standard treatment including hourly counseling sessions (M [SD] sessions attended 12 [8]). | |
|  |  |  | |  | |  | |
| Paris et al (2018) [51] | **CBT4CBT-Spanish + TAU.** A culturally adapted web-based treatment program that teaches core CBT skills for substance use disorders through modules, interactive exercises, quizzes, and more. The seven modules teach CBT skills via video vignettes that have been developed to align with Latino values. Skills taught include 1) understanding use patterns, 2) coping with cravings, 3) refusing substances, 4) developing problem-solving skills, 5) changing thinking about substances, and 6) improving decision-making skills. | 7 modules | | During treatment, participants could access the intervention as often as they wished (mean 5.3 modules completed). | | TAU included supportive counseling with access to other services (mean 5.2 group and 2.9 individual sessions completed). | |
|  |  |  | |  | |  | |
| Shi et al (2019) [143] | **CBT4CBT-Buprenophine + TAU.** A CBT-based intervention for buprenorphine maintenance among people with opioid use disorder. Eight modules teach CBT skills via videos, quizzes, and homework exercises. Videos cover buprenorphine information and treatment engagement. Skills taught include 1) understanding use patterns, 2) coping with cravings, 3) refusing substances, 4) developing problem-solving skills, 5) changing thinking about substances, and 6) improving decision-making skills. | 8 modules | | After the introductory module, participants could access modules at the clinic or at home (M [SD] modules completed 4.2 [2.0]). | | TAU included buprenorphine treatment and medication management (M days in protocol 69 of 84 total). | |
|  |  |  | |  | |  | |
| Schouten et al (2024) [56] | **Beating the Booze (BTB) + TAU**. A website intervention based on CBT and MI for comorbid depression and problematic alcohol use. Program focused on participants meeting reduction goals and recording alcohol use. Information conveyed through many mediums, including videos, patient stories, and interactive assignments. | 6 modules  (30-45 minutes per module) | | Modules were self-paced and could be completed across sessions (28.4% of participants reached last module). | | TAU for depression including evidence-based therapy and medication if necessary (94% completed 6-month assessment). | |
|  |  |  | |  | |  | |
| Takano et al (2022) [152] | **e-learning Serigaya Methamphetamine Relapse Prevention Program (e-SMARPP).** A web-based cognitive-behavioral relapse prevention program in which participants tracked their drug use and engaged with six relapse prevention sessions (each comprising three videos). Content included consequences of use, internal and external triggers, relapse prevention, patterns of thinking about use, leveraging social support, forming a crisis plan, and envisioning a future free of use. Users submitted homework after each session; homework was checked by therapists who gave personalized feedback. | 6 sessions  (25 min) | | Participants were told to submit daily use surveys by the end of each week (26.1% did not complete entire intervention). | | Web-based self-monitoring plus outpatient treatment (all participants completed entire intervention). | |
|  |  |  | |  | |  | |
| Tetrault et al (2020) [153] | **CBT4CBT + standard care.** The intervention is a web-based program that uses engaging video examples, animations, practice exercises, and narration to teach CBT. Skills taught include 1) understanding use patterns, 2) coping with cravings, 3) refusing substances, 4) developing problem-solving skills, 5) changing thinking about substances, and 6) improving decision-making skills. | 7 modules | | Participants were allowed to use the program whenever they liked (70% of participants completed all modules). | | Standard care included pharmacotherapy and counseling (86% completed week 8 follow-up). | |
|  |  |  |  | |  | |  |
| ***dCBI compared to CBT with therapist*** | |  |  | |  | |  |
| Johansson et al (2021) [109] | **Internet‐delivered CBT (ICBT).** An online intervention for alcohol use disorder based on MI, relapse prevention, and behavioral self‐control. Five main modules, with videos and short interviews with experts, covered 1) motivation, 2) drinking‐goal and self‐control, 3) behavioural analysis of drinking and risk‐situations, 4) general problem‐solving, and 5) preventing relapse. The program also included a use and craving diary, as well as asynchronous communication with a therapist who would remind participants to complete modules. | 5 main modules | | Participants told to spend 1-2 weeks per module (M modules completed 3.74). | | Participants learned module content via paper printouts and discussed it with therapists in-person (M modules completed 4.19). | |
|  |  |  | |  | |  | |
| Kay-Lambkin et al (2009) [113] comparison two | **Computer-delivered Self-Help for Alcohol and other drug use and Depression (SHADE) intensive therapy.** An online intervention based on CBT and MI for comorbid depression and alcohol/cannabis use disorder. In line with harm reduction, participants choose their therapy goals; content helps them explore the relationship between their substance use and their depression. Participants are shown video demonstrations, given exercises to learn CBT skills, and meet with a therapist post-session to review progress and schedule the next week’s appointment. | 9 sessions  (12 min) | | Participants told to complete nine sessions in sequence once a week (M [SD] sessions attended 7.61 [2.87]). | | Same material as tech intervention but delivered through in-person therapy (M [SD] sessions attended 8.71 [2.74]). | |
|  |  |  | |  | |  | |
| Kay-Lambkin et al (2011) [112] comparison two | **Clinician-assisted computerized [CAC] treatment.** An online intervention with minimal therapist guidance based on CBT and MI for comorbid depression and substance use. Participants completed computerized sessions followed by brief ten-minute individual check-ins with a trained psychotherapist. The first of nine sessions, for example, covered assessment feedback, psychoeducation about substance use and depression, and initial goal setting. | 9 sessions  (60 min allotted) | | Participants attended sessions once per week (29.9% received all sessions). | | Nine weekly hour-long sessions of CBT (34.1% received all sessions). | |
|  |  |  | |  | |  | |
| Kiluk et al (2018) [119] comparison two | **CBT4CBT.** An online intervention that includes voice-over narration, animations, quizzes, and other interactive elements to help teach CBT skills. Use of the program was supplemented by brief in-person check-ins with clinicians. Skills taught include 1) understanding use patterns, 2) coping with cravings, 3) refusing substances, 4) developing problem-solving skills, 5) changing thinking about substances, and 6) improving decision-making skills. | 7 modules | | Participants were asked to complete one module per week (M [SD] of modules was 5.5 [2.3]). | | Clinician-delivered CBT (12 individual sessions offered, M [SD] 4.1 [3.4]). | |
|  |  |  | |  | |  | |
| Tiburcio et al (2018) [154] comparison one | **Programa de Ayuda para Abuso de Drogas y Depresión (PAADD).** A computerized intervention based on the Transtheoretical Model of Change (TTM) and CBT targeting substance use and depression. The program teaches CBT skills such as substance use functional analysis, high risk situations, and action plans for coping as well as strategies to identify and restructure negative thoughts associated with depression. A counselor could be messaged through the intervention; they replied to queries with motivational feedback messages. | 8 weeks | | Participants could complete the intervention for at least one hour per week (M [SD] number of weeks logged in at least once 4.7 [2.2]). | | In-person CBT (M [SD] number of sessions 5.3 [2.0]). | |
|  |  |  | |  | |  | |
| Tiburcio et al (2018) [154] comparison two | **Programa de Ayuda para Abuso de Drogas y Depresión (PAADD).** A computerized intervention based on the Transtheoretical Model of Change (TTM) and CBT targeting substance use and depression. The program teaches CBT skills such as substance use functional analysis, high risk situations, and action plans for coping as well as strategies to identify and restructure negative thoughts associated with depression. A counselor could be messaged through the intervention; they replied to queries with motivational feedback messages. | 8 weeks | | Participants could complete the intervention for at least one hour per week (M [SD] number of weeks logged in at least once 4.7 [2.2]). | | ASSIST self-help guide followed by in-person CBT (M [SD] number of sessions 5.2 [1.6]). | |
|  |  |  |  | |  | |  |

*Notes. K =* 65. ACT = acceptance and commitment therapy, CBT = cognitive behavioral therapy, CBT4CBT = computer-based training for cognitive behavioral therapy, CM = contingency management, CRA = community reinforcement approach, DBT = dialectical behavioral therapy, MET = motivational enhancement therapy, MI = Motivational Interviewing, MMT = methadone maintenance treatment, TES = therapeutic enhancement system, TAU = treatment as usual, min. = minutes, M = mean, PBS = protective behavioral strategies, SD = standard deviation. ^1^ *Intervention dose (time to complete)* provides a measure of number of computer sessions, segments, or exercises, as described by study authors as well as a measure of time to complete each session, segment, or exercise. ^2^ *Flexibility (adherence)* provides information on whether the intervention was flexibly delivered, as needed to users and if reported, usage or adherence data are provided. ^3^ *Contrast (adherence)* provides a brief description of the contrast condition and if reported, usage or adherence data are provided.
