# Supplementary material for: Digitally Delivered Cognitive Behavioral Interventions for Alcohol and Other Drug Use: Meta-Analysis Across Consumption and Psychosocial Outcomes
Source: JMIR Ment Health. 2026 May 19;13:e82370. doi: 10.2196/82370 (PMC13231115; doi:10.2196/82370)
Supplement: Multimedia Appendix 3 [file mental_v13i1e82370_app3.docx]

**Table S1.** Measures and outcome scoring.

| **Measure** | **Item and Citation** | **Scoring Notes^1^** |
| --- | --- | --- |
| **Consumption: Abstinence Outcomes** | |  |
| Percent urine samples negative | Urine toxicology. |  |
| Number of weeks of consecutive abstinence | Urine toxicology. |  |
| Longest span of consecutive abstinence (days, OR weeks) | [1] |  |
| Percent participants abstinent (for week, OR for thirty days) | [1] |  |
| Percent participants relapsing | [1] | Reverse scored to harmonize direction across measures |
| Number of quit attempts | "The number of self-reported quit attempts lasting at least 24 hours" [2]. |  |
| **Consumption: Frequency Outcomes** | |  |
| Number of days abstinent (total, OR per week) | [1] |  |
| Number of days before relapse | [1] |  |
| Percent days abstinent | [1] |  |
| Use less than weekly across three months | [3] |  |
| Days of cocaine use | [1] | Reverse scored to harmonize direction across measures |
| Number of drinking OR substance use days (past week, OR past month) | [1] | Reverse scored to harmonize direction across measures |
| Number of drinking OR substance use days (past week, OR past month) | [4] | Reverse scored to harmonize direction across measures |
| Number of drinking OR substance use days (past week, OR past month) | [5] | Reverse scored to harmonize direction across measures |
| Number of drinking OR substance use days (past week, OR past month) | [6] | Reverse scored to harmonize direction across measures |
| Number of drinking OR substance use days (past week, OR past month) | [7] | Reverse scored to harmonize direction across measures |
| Number of drinking OR substance use days (past week, OR past month) | Detailed cannabis consumption tracker within app [8]. | Reverse scored to harmonize direction across measures |
| **Consumption: Heavy Frequency Outcomes** | |  |
| Number heavy drinking days | [1] | Reverse scored to harmonize direction across measures |
| Number heavy drinking days in past month | [7] | Reverse scored to harmonize direction across measures |
| Past-month hazardous use days | [9] | Reverse scored to harmonize direction across measures |
| Days drank to intoxication | [6] | Reverse scored to harmonize direction across measures |
| Percentage heavy drinking days | [10] | Reverse scored to harmonize direction across measures |
| **Consumption: Binge/Peak Outcomes** | |  |
| Average peak BAC per week (estimated using algorithm) | [11] | Reverse scored to harmonize direction across measures |
| Daily Drinking Questionnaire (DDQ) peak BAC | [4] | Reverse scored to harmonize direction across measures |
| Maximum single-day consumption | [7] | Reverse scored to harmonize direction across measures |
| Maximum single-day consumption | [5] | Reverse scored to harmonize direction across measures |
| Risky Single Occasion Drinking (RSOD) | "Over the past two weeks how many times did you consume more than four Australian standard drinks (ASDs; 10 g ethanol)? [in one sitting]" [12]. | Reverse scored to harmonize direction across measures |
| Number of days of "drunkness" within past month | "On how many days within the past 30 days did you feel drunk (e.g. unsteady on the feet, blurred vision, unclear speech?" [13]. | Reverse scored to harmonize direction across measures |
| Number of binge days | [4] | Reverse scored to harmonize direction across measures |
| Number of binge days | [1] | Reverse scored to harmonize direction across measures |
| Number of binge days | [5] | Reverse scored to harmonize direction across measures |
| Number of binge days within past month | [1] | Reverse scored to harmonize direction across measures |
| No excessive consumption | [4] |  |
| Percent participants excessive drinking | … | Reverse scored to harmonize direction across measures |
| **Consumption: Quantity Outcomes** | |  |
| Daily Drinking Questionnaire (DDQ) average estimated BAC | [4] | Reverse scored to harmonize direction across measures |
| Mean BAC | Log-transformed mean BAC per drinking day [14]. | Reverse scored to harmonize direction across measures |
| Drinks on drinking days | [1] | Reverse scored to harmonize direction across measures |
| Drinks on drinking days | [10] | Reverse scored to harmonize direction across measures |
| Grams of alcohol per day | [15] | Reverse scored to harmonize direction across measures |
| Drinks per day | [1] | Reverse scored to harmonize direction across measures |
| Drinks per week | [1] | Reverse scored to harmonize direction across measures |
| Drinks per week | [5] | Reverse scored to harmonize direction across measures |
| Drinks per week | [7] | Reverse scored to harmonize direction across measures |
| Drinks per week | [16] | Reverse scored to harmonize direction across measures |
| Drinks per week | [17] | Reverse scored to harmonize direction across measures |
| Drinks per week | [10] | Reverse scored to harmonize direction across measures |
| Drinks per month | [1] | Reverse scored to harmonize direction across measures |
| Grams of cannabis consumed in past week | [1] | Reverse scored to harmonize direction across measures |
| Joints consumed past week | Detailed cannabis consumption tracker within app [8]. | Reverse scored to harmonize direction across measures |
| Standard cannabis units per month | [1] | Reverse scored to harmonize direction across measures |
| Total quantity of cannabis | [1] | Reverse scored to harmonize direction across measures |
| Percentage of participants who changed to lower level of alcohol use | [18] |  |
| Reduction in weekly alcohol intake from baseline | Participants logged past-week drinking in consumption diary [19]. |  |
| Weekly quantity of cocaine use (grams, OR milligrams) | [1] | Reverse scored to harmonize direction across measures |
| **Consumption: Use Severity Outcomes** | |  |
| Alcohol Use Disorders Identification Test (AUDIT) | [18] | Reverse scored to harmonize direction across measures |
| Alcohol Use Disorders Identification Test-Consumption subscale (AUDIT-C) | [20] | Reverse scored to harmonize direction across measures |
| Cannabis Abuse Screening Test (CAST) | [21] | Reverse scored to harmonize direction across measures |
| Cannabis Use Disorders Identification Test-Revised (CUDIT-R) | [22] | Reverse scored to harmonize direction across measures |
| Drug Abuse Screening Test-20 (DAST-20) | [23] | Reverse scored to harmonize direction across measures |
| GAIN-I (Global Appraisal of Individual Needs - Initial) dependence subscale | [24] | Reverse scored to harmonize direction across measures |
| Number of self-reported DSM‐5 AUD criteria fulfilled | [25] | Reverse scored to harmonize direction across measures |
| Number of self-reported DSM‐5 CUD symptoms | [25] | Reverse scored to harmonize direction across measures |
| Number of self-reported ICD-10 alcohol dependence criteria | [26] | Reverse scored to harmonize direction across measures |
| Percent meeting DSM criteria for respective SUD | [27] | Reverse scored to harmonize direction across measures |
| Percent participants adhering to low-risk guidelines | [16] |  |
| Severity of Dependence Scale (SDS) | [28] | Reverse scored to harmonize direction across measures |
| **Consumption: Other Drug Use Outcomes** | |  |
| Alcohol, Smoking, Substance Involvement Screening Test (ASSIST) polydrug use | [3] | Reverse scored to harmonize direction across measures |
| Number of drinks in past week | [1] | Reverse scored to harmonize direction across measures |
| Number of drug use days | [1] | Reverse scored to harmonize direction across measures |
| Days of risky alcohol use in past 30 days | [29] | Reverse scored to harmonize direction across measures |
| Number of alcohol use days in past 30 days | [29] | Reverse scored to harmonize direction across measures |
| Did not binge drink within past month | [29] |  |
| Did not use alcohol within past month | [29] |  |
| Did not use cannabis within past month | [29] |  |
| **Psychosocial: Consequence Outcomes** | |  |
| Alcohol, Smoking, Substance Involvement Screening Test (ASSIST) stimulant score | [3] | Reverse scored to harmonize direction across measures |
| Brief-Young Adult Alcohol Consequences Questionnaire (B-YAACQ) | [30] | Reverse scored to harmonize direction across measures |
| Inventory of Drug Use Consequences (InDUC) | [31] | Reverse scored to harmonize direction across measures |
| Drinker’s Inventory of Consequences (DrInC) | [32] | Reverse scored to harmonize direction across measures |
| Problem Index | [33] | Reverse scored to harmonize direction across measures |
| Rutgers Alcohol Problem Index (RAPI) | [34] | Reverse scored to harmonize direction across measures |
| Current Opioid Misuse Measure (COMM) | [35] | Reverse scored to harmonize direction across measures |
| GAIN-I (Global Appraisal of Individual Needs - Initial) abuse score | [24] | Reverse scored to harmonize direction across measures |
| HIV Risk Taking Behavior Scale drug/sex risk total | [36] | Reverse scored to harmonize direction across measures |
| Percentage serious adverse events | "Serious adverse events are those resulting in death or leading to hospitalization." [37]. | Reverse scored to harmonize direction across measures |
| **Psychosocial: Cognitive Outcomes** | |  |
| Alcohol Abstinence Self-Efficacy Scale (AASE) | [38] |  |
| Self-efficacy Scale for Drug Dependence (SSDD) | [39] |  |
| Readiness to Change Questionnaire (RTCQ) percent of participants in "action" stage. | [40] |  |
| Stage of Change Readiness and Treatment Eagerness Scale-8 (SOCRATES) | [41] |  |
| Alcohol Expectancy Questionnaire (AEQ) | [42] | Reverse scored to harmonize direction across measures |
| Drinking Motives Questionnaire (DMQ-R-5) drinking motives, importance, confidence, and readiness subscales | [43] | Reverse scored to harmonize direction across measures |
| Penn Alcohol Craving Scale (PACS) | [44] | Reverse scored to harmonize direction across measures |
| Cocaine Craving Questionnaire-Brief (CCQ-Brief) | [45] | Reverse scored to harmonize direction across measures |
| Obsessive Compulsive Drinking Scale (OCDS) | [46] | Reverse scored to harmonize direction across measures |
| Stimulant Relapse Risk Scale (SRRS) | [47] | Reverse scored to harmonize direction across measures |
| Mental Help-Seeking Attitudes Scale (MHSAS) | [48] |  |
| **Psychosocial: Coping Outcomes** | | |
| Coping Strategies Scale (CSS) | [49] |  |
| Dialectical Behavior Therapy Ways of Coping Checklist (DBT-WCCL) | [50] |  |
| Mindful Attention Awareness Scale (MAAS) | [51] |  |
| Percent of participants who used CBT activities | Percentage of participants who endorsed (T/F) using a specific CBT activity to manage pain. All eight CBT activities were combined into a single percentage of endorsement [52]. |  |
| Direct Protective Behavioral Strategies (PBS) | [53] |  |
| Indirect Protective Behavioral Strategies (PBS) | [53] |  |
| Protective Behavioral Strategies (PBS) used to reduce drinking | "Over the past two weeks how many times did you use a PBS to control the amount of alcohol you drank?" Items were scored on a 4-point rating scale (0 = never, 1 = 1–2 times, 2 = 3–4 times, 3 = more than 4 times) [12]. |  |
| Protective Behavioral Strategies (PBS) used to reduce harm while drinking | "Over the past two weeks how many times did you use a PBS to reduce harm when drinking?" Items were scored on a 4-point rating scale (0 = never, 1 = 1–2 times, 2 = 3–4 times, 3 = more than 4 times) [12]. |  |
| Drug Risk Response Test (DRRT) overall response score | [54] |  |
| Drug Risk Response Test (DRRT) quality of overall response | [54] |  |
| Proportion of participants seeking formal help | [55] |  |
| Seeked professional help to reduce or end cannabis use since entering the study | … |  |
| **Psychosocial: Mental Health Outcomes** | |  |
| 16-item version of the Difficulty in Emotion Regulation Scale (DERS-16) | [56] | Reverse scored to harmonize direction across measures |
| Kessler Psychological Distress Scale (K-10) | [57] | Reverse scored to harmonize direction across measures |
| Mental Health Inventory (MHI-5) | [58] | Reverse scored to harmonize direction across measures |
| Beck Depression Inventory-II (BDI-11) | [59] | Reverse scored to harmonize direction across measures |
| Center for Epidemiologic Studies Depression Scale (CES-D) | [60] | Reverse scored to harmonize direction across measures |
| Patient Health Questionnaire-9 (PHQ-9) | [61] | Reverse scored to harmonize direction across measures |
| Montgomery Asberg Depression Rating Scale (MADRS‐S) | [62] | Reverse scored to harmonize direction across measures |
| Depression and Anxiety Stress Scale (DASS-21) | [63] | Reverse scored to harmonize direction across measures |
| Hospital Anxiety and Depression Scale (HADS) anxiety subscale | [64] | Reverse scored to harmonize direction across measures |
| Hospital Anxiety and Depression Scale (HADS) depression subscale | [64] | Reverse scored to harmonize direction across measures |
| Composite Social Interaction Anxiety Scale SF and Social Phobia Scale SF | [65] | Reverse scored to harmonize direction across measures |
| Generalized Anxiety Disorder–7 (GAD-7) | [66] | Reverse scored to harmonize direction across measures |
| PTSD Checklist–5 (PCL-5) | [67] | Reverse scored to harmonize direction across measures |
| PTSD Checklist–Military (PTSD-PCL) | [68] | Reverse scored to harmonize direction across measures |
| Scale for Suicidal Ideation (SSI) | [69] | Reverse scored to harmonize direction across measures |
| Suicidal Behaviors Questionnaire (SBQ) | [70] | Reverse scored to harmonize direction across measures |
| Attention Deficit and Hyperactivity Self-Report Scale version 1.1 (ASRS) | [71] | Reverse scored to harmonize direction across measures |
| **Psychosocial: General Health Outcomes** | |  |
| Log-transformed HIV viral load | … | Reverse scored to harmonize direction across measures |
| Physically unwell due to drinking | "Over the past two weeks how many times did you feel physically unwell due to your drinking?" Items were scored on a 4-point rating scale (0 = never, 1 = 1–2 times, 2 = 3–4 times, 3 = more than 4 times) [12]. | Reverse scored to harmonize direction across measures |
| Number of emergency department visits for pain | Participants asked to self-report number of times in last thirty days they visited the emergency department specifically for pain [52]. | Reverse scored to harmonize direction across measures |
| Pain Catastrophizing Scale (PCS) | [72] | Reverse scored to harmonize direction across measures |
| Multidimensional Pain Inventory (MPI) Pain Severity subscale | [73] | Reverse scored to harmonize direction across measures |
| Multidimensional Pain Inventory (MPI) Pain Interference subscale | [73] | Reverse scored to harmonize direction across measures |
| **Psychosocial: Quality of Life Outcomes** | |  |
| EQ-5D-5L VAS (Visual Analogue Scale) | [74] |  |
| European Health Interview Survey (EUROHIS) Quality of Life subscale | [75] |  |
| Five-dimensional EuroQol instrument (EQ-5D) | [76] |  |
| Flanagan Quality of Life Scale (QOLS) | [77] |  |
| Sense of Coherence Scale (SCS) | [78] |  |
| Sheehan Disability Scale (SDS) | [79] | Reverse scored to harmonize direction across measures |
| World Health Organization Disability Assessment Schedule 2.0 (WHODAS) | [80] | Reverse scored to harmonize direction across measures |
| Social Connectedness Scale (SCS) | [81] |  |
| Interpersonal issues due to drinking | "Over the past two weeks how many times did you experience interpersonal difficulties due to your drinking?" Items were scored on a 4-point rating scale (0 = never, 1 = 1–2 times, 2 = 3–4 times, 3 = more than 4 times) [12]. | Reverse scored to harmonize direction across measures |
| WHOQOL-BREF (Physical, Psychological, Social, and Environmental subscales) | [82] |  |
| Work/study consequences of drinking | "Over the past two weeks how many times did you experience difficulties with work and/or study due to your drinking?" Items were scored on a 4-point rating scale (0 = never, 1 = 1–2 times, 2 = 3–4 times, 3 = more than 4 times) [12]. | Reverse scored to harmonize direction across measures |
| **Other Outcomes** |  |  |
| Estimated alcohol use in excess using Opiate Treatment Index (OTI) quotient score | [9] | Reverse scored to harmonize direction across measures |
| Estimated cannabis use in excess using Opiate Treatment Index (OTI) quotient score | [9] | Reverse scored to harmonize direction across measures |

*Notes.* ^1^When calculating effect sizes, data were entered such that improvements in experimental treatment would yield a positive effect size across measures. As such, measures where higher scores indicated worse outcomes (such as increased binge drinking) were reverse-scored to harmonize directionality.

References for studies used in measures sheet.

[1] Sobell, L. C., & Sobell, M. B. (1992). Timeline follow-back: A technique for assessing self-reported alcohol consumption. In R. Z. Litten & J. P. Allen (Eds.), Measuring alcohol consumption: Psychosocial and biochemical methods (pp. 41–72). Humana Press/Springer Nature. https://doi.org/10.1007/978-1-4612-0357-5_3

[2] Olthof, M. I. A., Goudriaan, A. E., Van Laar, M. W., & Blankers, M. (2023). A guided digital intervention to reduce cannabis use: The ICan randomized controlled trial. Addiction, 118(9), 1775–1786. https://doi.org/10.1111/add.16217

[3] Humeniuk, R., Ali, R., Babor, T. F., Farrell, M., Formigoni, M. L., Jittiwutikarn, J., de Lacerda, R. B., Ling, W., Marsden, J., Monteiro, M., Nhiwatiwa, S., Pal, H., Poznyak, V., & Simon, S. (2008). Validation of the Alcohol, Smoking and Substance Involvement Screening Test (ASSIST). Addiction, 103(6), 1039–1047. https://doi.org/10.1111/j.1360-0443.2007.02114.x

[4] Collins, R. L., Parks, G. A., & Marlatt, G. A. (1985). Social determinants of alcohol consumption: the effects of social interaction and model status on the self-administration of alcohol. Journal of Consulting and Clinical Psychology, 53(2), 189–200. https://doi.org/10.1037//0022-006x.53.2.189

[5] Khadjesari, Z., Murray, E., Kalaitzaki, E., White, I. R., McCambridge, J., Godfrey, C., & Wallace, P. (2009). Test-retest reliability of an online measure of past week alcohol consumption (the TOT-AL), and comparison with face-to-face interview. Addictive Behaviors, 34(4), 337–342. https://doi.org/10.1016/j.addbeh.2008.11.010

[6] McLellan, A. T., Kushner, H., Metzger, D., Peters, R., Smith, I., Grissom, G., Pettinati, H., & Argeriou, M. (1992). The fifth edition of the Addiction Severity Index. Journal of Substance Abuse Treatment, 9(3), 199–213. https://doi.org/10.1016/0740-5472(92)90062-s

[7] Kruse M, Corbin W, Fromme K. Improving accuracy of QF measures of alcohol use: disaggregating quantity and frequency. Poster session presented at: 28th Annual Meeting of the Research Society on Alcoholism; 2005; Santa Barbara, CA.

[8] Schaub, M. P., Wenger, A., Berg, O., Beck, T., Stark, L., Buehler, E., & Haug, S. (2015). A web-based self-help intervention with and without chat counseling to reduce cannabis use in problematic cannabis users: Three-arm randomized controlled trial. Journal of Medical Internet Research, 17(10), e232. https://doi.org/10.2196/jmir.4860

[9] Darke, S., Heather, N., Hall, W., Ward, J., & Wodak, A. (1991). Estimating drug consumption in opioid users: reliability and validity of a 'recent use' episodes method. British Journal of Addiction, 86(10), 1311–1316. https://doi.org/10.1111/j.1360-0443.1991.tb01706.x

[10] Sobell, L. C., Agrawal, S., Sobell, M. B., Leo, G. I., Young, L. J., Cunningham, J. A., & Simco, E. R. (2003). Comparison of a quick drinking screen with the timeline followback for individuals with alcohol problems. Journal of Studies on Alcohol, 64(6), 858–861. https://doi.org/10.15288/jsa.2003.64.858

[11] Markham, M.R., Miller, W.R. & Arciniega, L. BACCuS 2.01: Computer software for quantifying alcohol consumption. Behavior Research Methods, Instruments, & Computers 25, 420–421 (1993). https://doi.org/10.3758/BF03204538

[12] O’Donnell, R., Richardson, B., Fuller-Tyszkiewicz, M., & Staiger, P. K. (2019). Delivering personalized protective behavioral drinking strategies via a smartphone intervention: A pilot study. International Journal of Behavioral Medicine, 26(4), 401–414. https://doi.org/10.1007/s12529-019-09789-0

[13] Zill, J. M., Christalle, E., Meyer, B., Härter, M., & Dirmaier, J. (2019). The effectiveness of an internet intervention aimed at reducing alcohol consumption in adults. Deutsches Ärzteblatt International, 116, 127–133. https://doi.org/10.3238/arztebl.2019.0127

[14] Hester, R. K., Delaney, H. D., & Campbell, W. (2011). ModerateDrinking.Com and moderation management: Outcomes of a randomized clinical trial with non-dependent problem drinkers. Journal of Consulting and Clinical Psychology, 79(2), 215–224. https://doi.org/10.1037/a0022487

[15] Kraus, L., Piontek, D., Pabst, A., & Matos, E. G. de. (2013). Studiendesign und methodik des epidemiologischen suchtsurveys 2012. SUCHT, 59(6), 309–320. https://doi.org/10.1024/0939-5911.a000274

[16] Lemmens, P., Knibbe, R. A., & Tan, F. (1988). Weekly recall and dairy estimates of alcohol consumption in a general population survey. Journal of Studies on Alcohol, 49(2), 131–135. https://doi.org/10.15288/jsa.1988.49.131

[17] Miller WR, Marlatt GA. The Brief Drinker Profile. Odessa, FL: Psychological Assessment Resources; 1987.

[18] Saunders, J. B., Aasland, O. G., Babor, T. F., de la Fuente, J. R., & Grant, M. (1993). Development of the Alcohol Use Disorders Identification Test (AUDIT): WHO collaborative project on early detection of persons with harmful alcohol consumption--II. Addiction, 88(6), 791–804. https://doi.org/10.1111/j.1360-0443.1993.tb02093.x

[19] Guillemont, J., Cogordan, C., Nalpas, B., Nguyen-Thanh, V., Richard, J.-B., & Arwidson, P. (2017). Effectiveness of a web-based intervention to reduce alcohol consumption among French hazardous drinkers: A randomized controlled trial. Health Education Research, 32(4), 332–342. https://doi.org/10.1093/her/cyx052

[20] Dawson, D. A., Grant, B. F., Stinson, F. S., & Zhou, Y. (2005). Effectiveness of the derived Alcohol Use Disorders Identification Test (AUDIT-C) in screening for alcohol use disorders and risk drinking in the US general population. Alcoholism, clinical and experimental research, 29(5), 844–854. https://doi.org/10.1097/01.alc.0000164374.32229.a2

[21] Legleye, S., Karila, L., Beck, F., & Reynaud, M. (2007). Validation of the CAST, a general population Cannabis Abuse Screening Test. Journal of Substance Use, 12(4), 233–242. https://doi.org/10.1080/14659890701476532

[22] Annaheim, B., Scotto, T. J., & Gmel, G. (2010). Revising the Cannabis Use Disorders Identification Test (CUDIT) by means of Item Response Theory. International journal of methods in psychiatric research, 19(3), 142–155. https://doi.org/10.1002/mpr.308

[23] Skinner H. A. (1982). The drug abuse screening test. Addictive Behaviors, 7(4), 363–371. https://doi.org/10.1016/0306-4603(82)90005-3

[24] Dennis, M. L., Chan, Y. F., & Funk, R. R. (2006). Development and validation of the GAIN Short Screener (GSS) for internalizing, externalizing and substance use disorders and crime/violence problems among adolescents and adults. The American Journal on Addictions, 15 Suppl 1(Suppl 1), 80–91. https://doi.org/10.1080/10550490601006055

[25] American Psychiatric Association. (2013). Diagnostic and statistical manual of mental disorders (5th ed.). https://doi.org/10.1176/appi.books.9780890425596

[26] World Health Organization. (1992). The ICD-10 Classification of mental and behavioural disorders: clinical descriptions and diagnostic guidelines (10th ed.). ISBN: 978-9241544221

[27] American Psychiatric Association. (2000). Diagnostic and statistical manual of mental disorders (4th ed., text rev.). ISBN: 978-0890420256

[28] Gossop, M., Darke, S., Griffiths, P., Hando, J., Powis, B., Hall, W., & Strang, J. (1995). The Severity of Dependence Scale (SDS): psychometric properties of the SDS in English and Australian samples of heroin, cocaine and amphetamine users. Addiction, 90(5), 607–614. https://doi.org/10.1046/j.1360-0443.1995.9056072.x

[29] Kokkevi, A., Hartgers, C. (1995). EuropASI: European adaptation of a multidimensional assessment instrument for drug and alcohol dependence. European Addiction Research, 1(4), 208–210. https://doi.org/10.1159/000259089

[30] Kahler, C. W., Strong, D. R., & Read, J. P. (2005). Toward efficient and comprehensive measurement of the alcohol problems continuum in college students: The Brief Young Adult Alcohol Consequences Questionnaire. Alcoholism: Clinical and Experimental Research, 29(7), 1180-1189. https://doi.org/10.1097/01.alc.0000171940.95813.a5

[31] Tonigan, J. S., & Miller, W. R. (2002). The inventory of drug use consequences (InDUC): test-retest stability and sensitivity to detect change. Psychology of Addictive Behaviors, 16(2), 165. https://doi.org/10.1037/0893-164X.16.2.165

[32] Miller WR, Tonigan JS, Longabaugh R, Mattson ME. The Drinker Inventory of Consequences (DrInC). Rockville, MD: National Institute on Alcohol Abuse and Alcoholism; 1995.

[33] Cornel, M., Knibbe, R. A., van Zutphen, W. M., & Drop, M. J. (1994). Problem drinking in a general practice population: The construction of an interval scale for severity of problem drinking. Journal of Studies on Alcohol, 55, 466−470. https://doi.org/10.15288/jsa.1994.55.466

[34] White, H. R., & Labouvie, E. W. (1989). Towards the assessment of adolescent problem drinking. Journal of Studies on Alcohol, 50(1), 30–37. https://doi.org/10.15288/jsa.1989.50.30

[35] Butler, S. F., Budman, S. H., Fernandez, K. C., Houle, B., Benoit, C., Katz, N., & Jamison, R. N. (2007). Development and validation of the Current Opioid Misuse Measure. Pain, 130(1-2), 144–156. https://doi.org/10.1016/j.pain.2007.01.014

[36] Darke, S., Hall, W., Heather, N., Ward, J., & Wodak, A. (1991). The reliability and validity of a scale to measure HIV risk-taking behaviour among intravenous drug users. AIDS, 5(2), 181–185. https://doi.org/10.1097/00002030-199102000-00008

[37] Kiluk, B. D., Nich, C., Buck, M. B., Devore, K. A., Frankforter, T. L., LaPaglia, D. M., Muvvala, S. B., & Carroll, K. M. (2018). Randomized clinical trial of computerized and clinician-delivered CBT in comparison with standard outpatient treatment for substance use disorders: Primary within-treatment and follow-up outcomes. American Journal of Psychiatry, 175(9), 853–863. https://doi.org/10.1176/appi.ajp.2018.17090978

[38] DiClemente, C. C., Carbonari, J. P., Montgomery, R. P., & Hughes, S. O. (1994). The Alcohol Abstinence Self-Efficacy scale. Journal of Studies on Alcohol, 55(2), 141–148. https://doi.org/10.15288/jsa.1994.55.141

[39] Morita, N., Suetsugu, S., Shimane, T., Okasaka, Y., Kiyoshige, T., & Iwai, K. (2007). Japanese Journal of Alcohol Studies & Drug Dependence, 42(5), 487–506. PMID: 18051469

[40] Rollnick, S., Heather, N., Gold, R., & Hall, W. (1992). Development of a short ‘readiness to change’ questionnaire for use in brief, opportunistic interventions among excessive drinkers. British Journal of Addiction, 87(5), 743-754. https://doi.org/10.1111/j.1360-0443.1992.tb02720.x

[41] Miller, W. R., & Tonigan, J. S. (1996). Assessing drinkers' motivation for change: The Stages of Change Readiness and Treatment Eagerness Scale (SOCRATES). Psychology of Addictive Behaviors, 10(2), 81–89. https://doi.org/10.1037/0893-164X.10.2.81

[42] Brown, S. A., Christiansen, B. A., & Goldman, M. S. (1987). The Alcohol Expectancy Questionnaire: an instrument for the assessment of adolescent and adult alcohol expectancies. Journal of Studies on Alcohol, 48(5), 483–491. https://doi.org/10.15288/jsa.1987.48.483

[43] Cooper, M. L. (1994). Motivations for alcohol use among adolescents: Development and validation of a four-factor model. Psychological Assessment, 6(2), 117–128. https://doi.org/10.1037/1040-3590.6.2.117

[44] Flannery, B. A., Volpicelli, J. R., & Pettinati, H. M. (1999). Psychometric properties of the Penn alcohol craving scale. Alcoholism: Clinical and Experimental Research, 23(8), 1289e1295. https://doi.org/10.1111/j.1530-0277.1999.tb04349.x

[45] Sussner, B. D., Smelson, D. A., Rodrigues, S., Kline, A., Losonczy, M., & Ziedonis, D. (2006). The validity and reliability of a brief measure of cocaine craving. Drug and Alcohol Dependence, 83(3), 233–237. https://doi.org/10.1016/j.drugalcdep.2005.11.022

[46] Anton, R. F., Moak, D. H., & Latham, P. K. (1996). The obsessive compulsive drinking scale: a new method of assessing outcome in alcoholism treatment studies. Archives of General Psychiatry, 53(3), 225-231. https://doi.org/10.1001/archpsyc.1996.01830030047008

[47] Ogai, Y., Haraguchi, A., Kondo, A., Ishibashi, Y., Umeno, M., Kikumoto, H., Hori, T., Komiyama, T., Kato, R., Aso, K., Asukai, N., Senoo, E., & Ikeda, K. (2007). Development and validation of the Stimulant Relapse Risk Scale for drug abusers in Japan. Drug and Alcohol Dependence, 88(2-3), 174–181. https://doi.org/10.1016/j.drugalcdep.2006.10.005

[48] Hammer, J. H., Parent, M. C., & Spiker, D. A. (2018). Mental Help Seeking Attitudes Scale (MHSAS): Development, reliability, validity, and comparison with the ATSPPH-SF and IASMHS-PO. Journal of Counseling Psychology, 65(1), 74–85. https://doi.org/10.1037/cou0000248

[49] Litt, M. D., Kadden, R. M., Cooney, N. L., & Kabela, E. (2003). Coping skills and treatment outcomes in cognitive-behavioral and interactional group therapy for alcoholism. Journal of Consulting and Clinical Psychology, 71(1), 118–128. https://doi.org/10.1037//0022-006x.71.1.118

[50] Neacsiu, A. D., Rizvi, S. L., Vitaliano, P. P., Lynch, T. R., & Linehan, M. M. (2010). The dialectical behavior therapy ways of coping checklist: development and psychometric properties. Journal of Clinical Psychology, 66(6), 563–582. https://doi.org/10.1002/jclp.20685

[51] Brown, K. W., & Ryan, R. M. (2003). The benefits of being present: Mindfulness and its role in psychological well-being. Journal of Personality and Social Psychology, 84(4), 822–848. https://doi.org/10.1037/0022-3514.84.4.822

[52] Guarino, H., Fong, C., Marsch, L. A., Acosta, M. C., Syckes, C., Moore, S. K., Cruciani, R. A., Portenoy, R. K., Turk, D. C., & Rosenblum, A. (2018). Web-based cognitive behavior therapy for chronic pain patients with aberrant drug-related behavior: Outcomes from a randomized controlled trial. Pain Medicine, 19(12), 2423–2437. https://doi.org/10.1093/pm/pnx334

[53] Palmer RS. Efficacy of the alcohol skills training program in mandated and nonmandated heavy drinking college students. Dissertation. 2004. Dissertation Abstracts International: Section B: The Sciences and Engineering. 65(5-B):2644.

[54] Kiluk, B. D., Nich, C., Babuscio, T., & Carroll, K. M. (2010). Quality versus quantity: acquisition of coping skills following computerized cognitive-behavioral therapy for substance use disorders. Addiction, 105(12), 2120–2127. https://doi.org/10.1111/j.1360-0443.2010.03076.x

[55] Bouwmans, C., De Jong, K., Timman, R., Zijlstra-Vlasveld, M., Van der Feltz-Cornelis, C., Tan Swan, S., & Hakkaart-van Roijen, L. (2013). Feasibility, reliability and validity of a questionnaire on healthcare consumption and productivity loss in patients with a psychiatric disorder (TiC-P). BMC health services research, 13, 217. https://doi.org/10.1186/1472-6963-13-217

[56] Gratz, K. L., & Roemer, L. (2004). Multidimensional assessment of emotion regulation and dysregulation: Development, factor structure, and initial validation of the difficulties in emotion regulation scale. Journal of Psychopathology and Behavioral Assessment, 26, 41-54. https://doi.org/10.1007/s10862-008-9102-4

[57] Kessler, R. C., Andrews, G., Colpe, L. J., Hiripi, E., Mroczek, D. K., Normand, S. L., Walters, E. E., & Zaslavsky, A. M. (2002). Short screening scales to monitor population prevalences and trends in non-specific psychological distress. Psychological Medicine, 32(6), 959–976. https://doi.org/10.1017/s0033291702006074

[58] Rumpf, H. J., Meyer, C., Hapke, U., & John, U. (2001). Screening for mental health: validity of the MHI-5 using DSM-IV Axis I psychiatric disorders as gold standard. Psychiatry Research, 105(3), 243–253. https://doi.org/10.1016/s0165-1781(01)00329-8

[59] Beck, A. T., Steer, R. A., and Brown, G. K. (1996). Manual for the Beck Depression Inventory-II. San Antonio, TX: Psychological Corporation. https://doi.org/10.1037/t00742-000

[60] Cole, J. C., Rabin, A. S., Smith, T. L., & Kaufman, A. S. (2004). Development and validation of a Rasch-derived CES-D short form. Psychological Assessment, 16(4), 360–372. https://doi.org/10.1037/1040-3590.16.4.360

[61] Kroenke, K., & Spitzer, R. L. (2002). The PHQ-9: a new depression diagnostic and severity measure. Psychiatric Annals, 32(9), 509-515. https://doi.org/10.3928/0048-5713-20020901-06

[62] Svanborg, P., & Asberg, M. (2001). A comparison between the Beck Depression Inventory (BDI) and the self-rating version of the Montgomery Asberg Depression Rating Scale (MADRS). Journal of Affective Disorders, 64(2-3), 203–216. https://doi.org/10.1016/s0165-0327(00)00242-1

[63] Lovibond, P. F., & Lovibond, S. H. (1995). The structure of negative emotional states: comparison of the Depression Anxiety Stress Scales (DASS) with the Beck Depression and Anxiety Inventories. Behaviour research and therapy, 33(3), 335–343. https://doi.org/10.1016/0005-7967(94)00075-u

[64] Zigmond, A. S., & Snaith, R. P. (1983). The hospital anxiety and depression scale. Acta Psychiatrica Scandinavica, 67(6), 361–370. https://doi.org/10.1111/j.1600-0447.1983.tb09716.x

[65] Peters, L., Sunderland, M., Andrews, G., Rapee, R. M., & Mattick, R. P. (2012). Development of a short form Social Interaction Anxiety (SIAS) and Social Phobia Scale (SPS) using nonparametric item response theory: the SIAS-6 and the SPS-6. Psychological Assessment, 24(1), 66–76. https://doi.org/10.1037/a0024544

[66] Löwe, B., Decker, O., Müller, S., Brähler, E., Schellberg, D., Herzog, W., & Herzberg, P. Y. (2008). Validation and standardization of the Generalized Anxiety Disorder Screener (GAD-7) in the general population. Medical Care, 46(3), 266–274. https://doi.org/10.1097/MLR.0b013e318160d093

[67] Weathers FW, Litz BT, Keane TM, Palmieri PA, Marx BP, Schnurr PP. The PTSD Checklist for DSM-5 (PCL-5). National Center for PTSD; 2010.

[68] Weather, F., & Ford, J. (1996). Psychometric review of PTSD checklist (PCL-C, PCL-S, PCL-M, PCL-PR). In B. Stamm (Ed.). Measurement of stress, trauma, and adaptation (pp. 250-251). Lutherville, MD: Sidran Press. https://doi.org/10.1080/00029157.2000.10734366

[69] Beck, A. T., Kovacs, M., & Weissman, A. (1979). Assessment of suicidal intention: the Scale for Suicide Ideation. Journal of Consulting and Clinical Psychology, 47(2), 343–352. https://doi.org/10.1037//0022-006x.47.2.343

[70] Osman, A., Bagge, C. L., Gutierrez, P. M., Konick, L. C., Kopper, B. A., & Barrios, F. X. (2001). The Suicidal Behaviors Questionnaire-Revised (SBQ-R): Validation with clinical and nonclinical samples. Assessment, 8(4), 443-454. https://doi.org/10.1177/107319110100800409

[71] Daigre Blanco, C., Ramos-Quiroga, J. A., Valero, S., Bosch, R., Roncero, C., Gonzalvo, B., & Nogueira, M. (2009). Adult ADHD Self-Report Scale (ASRS-v1.1) symptom checklist in patients with substance use disorders. Actas Espanolas de Psiquiatria, 37(6), 299–305. PMID: 20066581

[72] Sullivan, M. J. L., Bishop, S. R., & Pivik, J. (1995). The Pain Catastrophizing Scale: Development and validation. Psychological Assessment, 7(4), 524–532. https://doi.org/10.1037/1040-3590.7.4.524

[73] Kerns, R. D., Turk, D. C., & Rudy, T. E. (1985). The West Haven-Yale Multidimensional Pain Inventory (WHYMPI). Pain, 23(4), 345–356. https://doi.org/10.1016/0304-3959(85)90004-1

[74] Herdman, M., Gudex, C., Lloyd, A., Janssen, M., Kind, P., Parkin, D., Bonsel, G., & Badia, X. (2011). Development and preliminary testing of the new five-level version of EQ-5D (EQ-5D-5L). Quality of Life Research, 20(10), 1727–1736. https://doi.org/10.1007/s11136-011-9903-x

[75] Schmidt, S., Mühlan, H., & Power, M. (2006). The EUROHIS-QOL 8-item index: psychometric results of a cross-cultural field study. European journal of public health, 16(4), 420–428. https://doi.org/10.1093/eurpub/cki155

[76] EuroQol Group. (1990). EuroQol: A new facility for the measurement of health-related quality of life. Health Policy, 16, 199–208. https://doi.org/10.1016/0168-8510(90)90421-9

[77] Flanagan, J. C. (1978). A research approach to improving our quality of life. American Psychologist, 33(2), 138. https://doi.org/10.1037/0003-066X.33.2.138

[78] Lundqvist T. (1995). Chronic cannabis use and the sense of coherence. Life Sciences, 56(23-24), 2145–2150. https://doi.org/10.1016/0024-3205(95)00201-g

[79] Sheehan, D. V. (2000). Sheehan disability scale. Handbook of psychiatric measures. ISBN: 9780890424155

[80] Ustün, T. B., Chatterji, S., Kostanjsek, N., Rehm, J., Kennedy, C., Epping-Jordan, J., Saxena, S., von Korff, M., Pull, C., & WHO/NIH Joint Project (2010). Developing the World Health Organization Disability Assessment Schedule 2.0. Bulletin of the World Health Organization, 88(11), 815–823. https://doi.org/10.2471/BLT.09.067231

[81] Lee, R. M., & Robbins S. B. (1995). Measuring belongingness: The social connectedness and the social assurance scales. Journal of Counseling Psychology, 42, 232-241. https://doi.org/10.1037/0022-0167.42.2.232

[82] Skevington, S.M., Lofty, M., O'Connell, K.A., & WHOQOL Group (2004). The WHOQOL-BREF quality of life assessment; psychometric properties and results of the international field trial: a report from the WHOQOL group. Quality of Life Research, 13, 299–310. https://doi.org/10.1023/B:QURE.0000018486.91360.00
