# Supplementary figures and images for: Digitally Delivered Cognitive Behavioral Interventions for Alcohol and Other Drug Use: Meta-Analysis Across Consumption and Psychosocial Outcomes
Source: JMIR Ment Health. 2026 May 19;13:e82370. doi: 10.2196/82370 (PMC13231115; doi:10.2196/82370)

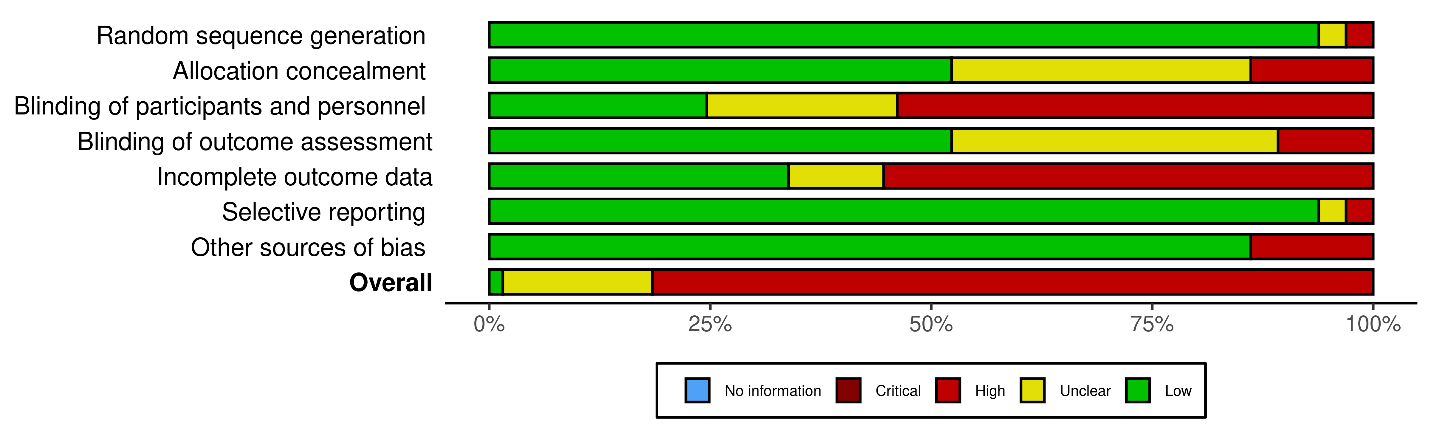


**Figure S1.** Sample-level risk of bias assessment.

Supplement: Multimedia Appendix 5 [file mental_v13i1e82370_app5.docx]
