# Supplementary material for: Digitally Delivered Cognitive Behavioral Interventions for Alcohol and Other Drug Use: Meta-Analysis Across Consumption and Psychosocial Outcomes
Source: JMIR Ment Health. 2026 May 19;13:e82370. doi: 10.2196/82370 (PMC13231115; doi:10.2196/82370)
Supplement: Multimedia Appendix 6 [file mental_v13i1e82370_app6.docx]

| Pooled effect size type | K | Hedges's *g* | Lower CI | Upper CI | *p* | I^2^ |
| --- | --- | --- | --- | --- | --- | --- |
| Between groups combined |  |  |  |  |  |  |
| **Consumption effect size** | **63** | **0.22** | **0.15** | **0.30** | **< .001** | **68.68** |
| **Psychosocial effect size** | **39** | **0.13** | **0.05** | **0.21** | **.001** | **46.04** |
| Minimal treatment |  |  |  |  |  |  |
| **Consumption effect size** | **31** | **0.27** | **0.16** | **0.38** | **< .001** | **79.17** |
| **Psychosocial effect size** | **16** | **0.14** | **0.04** | **0.24** | **.005** | **41.23** |
| Another treatment |  |  |  |  |  |  |
| Consumption effect size | 12 | 0.08 | -0.15 | 0.31 | .504 | 78.40 |
| Psychosocial effect size | 10 | 0.18 | -0.06 | 0.41 | .138 | 76.96 |
| Add-on to usual treatment |  |  |  |  |  |  |
| **Consumption effect size** | **20** | **0.23** | **0.15** | **0.32** | **< .001** | **0.00** |
| Psychosocial effect size | 12 | 0.12 | -0.03 | 0.28 | .126 | 30.91 |
| Therapist-delivered CBI |  |  |  |  |  |  |
| Consumption effect size | 5 | 0.20 | -0.08 | 0.48 | .165 | 0.59 |
| Psychosocial effect size | 5 | -0.03 | -0.19 | 0.14 | .763 | 0.00 |
| Within-group (baseline) |  |  |  |  |  |  |
| **Consumption effect size** | **45** | **0.70** | **0.62** | **0.81** | **< .001** | **89.47** |
| **Psychosocial effect size** | **36** | **0.52** | **0.41** | **0.63** | **< .001** | **89.39** |

**Table S1.** Meta-analysis with study effect sizes averaged to the study-level.

*Notes:* Pooled estimates significant at *a =* .05 in **bold**.
