# Supplementary material for: Digitally Delivered Cognitive Behavioral Interventions for Alcohol and Other Drug Use: Meta-Analysis Across Consumption and Psychosocial Outcomes
Source: JMIR Ment Health. 2026 May 19;13:e82370. doi: 10.2196/82370 (PMC13231115; doi:10.2196/82370)
Supplement: Multimedia Appendix 7 [file mental_v13i1e82370_app7.docx]

**Figure S1.** Plot of assessment of publication bias.

*Note.* Assessment of bias in consumption outcomes for dCBI effect in contrast to a minimal intervention control. The plot shows symmetry, and the rank order correlation shows a non-significant association between precision and effect size (τ = 0.17, *p* > .05).

**Figure S2.** Plot of assessment of publication bias.

*Note.* Assessment of bias in psychosocial outcomes for dCBI effect in contrast to a minimal intervention control. The plot shows symmetry, and the rank order correlation shows a non-significant association between precision and effect size (τ = 0.28, *p* > .05).

**Figure S3.** Plot of assessment of publication bias.

*Note.* Assessment of bias in consumption outcomes for dCBI effect in contrast to another intervention. The plot shows symmetry with one high-negative effect, low precision study (O’Donnell et al., 2019), and the rank order correlation shows a non-significant association between precision and effect size (τ = -0.06, *p* > .05).

**Figure S4.** Plot of assessment of publication bias.

*Note.* Assessment of bias in psychosocial outcomes for dCBI effect in contrast to another intervention. The plot shows symmetry with one high-positive effect, low precision study (O’Donnell et al., 2019), and the rank order correlation shows a non-significant association between precision and effect size (τ = 0.02, *p* > .05).

**Figure S5.** Plot of assessment of publication bias.

*Note.* Assessment of bias in consumption outcomes for dCBI effect when added to usual care and contrasted with usual care alone. The plot shows symmetry, and the rank order correlation shows a non-significant association between precision and effect size (τ = 0.08, *p* > .05).

**Figure S6.** Plot of assessment of publication bias.

*Note.* Assessment of bias in psychosocial outcomes for dCBI effect when added to usual care and contrasted with usual care alone. The plot shows symmetry, and the rank order correlation shows a non-significant association between precision and effect size (τ = 0.30, *p* > .05).

**Figure S7.** Plot of assessment of publication bias.

*Note.* Assessment of bias in consumption outcomes for dCBI effect when contrasted with CBI delivered in person. The plot shows symmetry, and the rank order correlation shows a non-significant association between precision and effect size (τ = 0.40, *p* > .05).

**Figure S8.** Plot of assessment of publication bias.

*Note.* Assessment of bias in psychosocial outcomes for dCBI effect when contrasted with CBI delivered in person. The plot shows symmetry, and the rank order correlation shows a non-significant association between precision and effect size (τ = 0.60, *p* > .05).
